# Rapid Fabrication of Self‐Propelled and Steerable Magnetic Microcatheters for Precision Medicine

**DOI:** 10.1002/adma.202506591

**Published:** 2025-11-27

**Authors:** Zhi Chen, Boris Rivkin, David Castellanos‐Robles, Ivan Soldatov, Lukas Beyer, Mariana Medina‐Sánchez

**Affiliations:** ^1^ CIC nanoGUNE 20018 San Sebastián Spain; ^2^ Leibniz Institute for Solid State and Materials Research (IFW) 01069 Dresden Germany; ^3^ Center for Molecular Bioengineering (B CUBE) Dresden University of Technology 01307 Dresden Germany; ^4^ Institute of Materials Science Technische Universität Bergakademie Freiberg 09599 Freiberg Germany; ^5^ IKERBASQUE Basque Foundation for Science 48009 Bilbao Spain

**Keywords:** assisted reproduction technologies (ART), intracorporeal navigation, magnetic soft continuum robot, mass produced tubular devices, microrobotic platform, minimally invasive therapies, self‐propelled microcatheter, targeted drug delivery systems, tracheal intervention, wave‐crawling locomotion

## Abstract

Minimally invasive therapies demand precise navigation through complex and delicate anatomical pathways, requiring medical tools that are small, flexible, and highly maneuverable. Here, a high‐yield fabrication method for the production of magnetic tubular microrobots, tethered and untethered, with programmable magnetization, is presented. The method uses Joule heating through a template wire, enabling the fabrication of microrobots with tunable dimensions. Three device configurations are demonstrated: 1) a steerable guiding microcatheter with stiffness modulation; 2) an untethered tubular microrobot (TubeBot), exhibiting wave‐crawling locomotion; and 3) a hybrid microcatheter robot that integrates distal‐end wave‐crawling propulsion with linear insertion to minimize tissue trauma. Validation in tortuous channels, soft phantoms replicating tissue compliance, 3D‐printed organ models, ex vivo tissues, and live mice demonstrates the microrobots's ability to achieve precise navigation across different environments. The successful targeted delivery of sperm cells, embryos, and drug‐mimicking compounds further highlights its potential for precision medicine, including applications in assisted reproduction and targeted drug delivery.

## Graphical abstract

A rapid Joule heating fabrication method for the production of self‐propelling, adaptive microcatheters, with tunable stiffness and integrated microfluidic channels is presented. Demonstrated through three microrobotic designs, including a steerable guiding catheter, an untethered wave‐crawling TubeBot, and a distal‐end propelled microcatheter, it was possible to achieve robust navigation in confined, delicate environments, validated in diverse synthetic and biological models, offering new possibilities for targeted intracorporeal interventions.

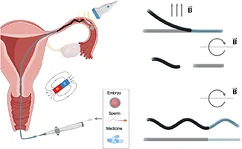

## 1 Introduction

In modern medicine, minimally invasive therapies are essential, providing reduced trauma, faster recovery, and fewer side effects.^[^ ^1^ ^]^ However, conventional tools for these procedures, such as laparoscopic instruments, guidewires, and catheters, typically range from millimeters to centimeters in diameter and often lack adaptive maneuverability, limiting their effectiveness, particularly in hard‐to‐reach regions of the body.^[^ ^2^, ^3^, ^4^ ^]^ Recent efforts have been focused on optimizing these devices for mechanical performance, miniaturization, and functionality, while minimizing tissue damage.^[^ ^5^, ^6^ ^]^ For instance, in reproductive medicine, falloposcopy has made significant advances in miniaturization, enabling direct visualization of the fallopian tubes using tiny flexible endoscopes. This allows detection of tubal abnormalities, assessment of the mucosal lining, and the performance of minor therapeutic interventions, such as clearing small obstructions.^[^ ^7^, ^8^, ^9^, ^10^ ^]^ However, these devices remain largely passive, relying on mechanical pushing from outside the body. They tend to scrape the fallopian tube cilia during insertion, and face challenges introducing them into the complex and variable anatomy of the fallopian tubes (see Table S1 in the Supporting Information for a comparative overview). Hysterosalpingography (HSG) another method used for reproductive medicine to assess the utherus and fallopian tubes. It is employs fluoroscopy for imaging, in which a contrast dye is injected via a catheter. However, it is primarily used for diagnostic purposes, during which X‐ray exposure is minimal (approximately 1–2 minutes). Prolonged or repeated radiation exposure, on the other hand, may lead to adverse effects.^[^ ^11^, ^12^ ^]^ Similarly, procedures such as artificial insemination near the fertilization site, intrafallopian embryo transfer, and endometriosis/cancer management often rely on laparoscopy, which requires multiple instruments for imaging, illumination, and surgical intervention.^[^ ^13^, ^14^, ^15^, ^16^, ^17^ ^]^ These tools are relatively large and can increase the risk of tissue injury, infection, or, in some cases, ectopic pregnancies.^[^ ^13^, ^18^ ^]^ Comparable challenges exist in other biomedical scenarios, such as the peripheral microcirculation or terminal bronchioles and alveolar sacs in the lungs.^[^ ^4^, ^19^ ^]^

In this context, continuum robots (CRs) offer a promising solution. Their structures bend continuously along their length, enabling navigation through tortuous anatomical pathways while remaining connected to an external control hardware.^[^ ^20^ ^]^ CRs have been applied successfully in clinical procedures including cardiac ablation, lung biopsy, and transoral robotic surgery.^[^ ^21^, ^22^, ^23^ ^]^ But they are still large in size (mm to cm range), and typically rely on tendon‐like, hydraulic, shape‐memory alloy, or polymer‐based mechanisms for distal‐tip manipulation (Table S2, Supporting Information).^[^ ^24^, ^25^, ^26^, ^27^, ^28^, ^29^ ^]^ A strategy to miniaturize the CRs further, is by incorporating magnetic materials into flexible segments to enable their remote actuation using global magnetic fields.^[^ ^30^ ^]^ Replacing rigid permanent magnets with micro‐ or nanoparticles further alleviates size constraints but their weaker magnetic moments require stronger external fields to achieve effective actuation.^[^ ^31^ ^]^ These challenges highlight the need for novel actuation strategies to enhance navigational performance in intricated organs.^[^ ^32^, ^33^, ^34^, ^35^ ^]^ Untethered microrobots provide a possible solution, enabling remote actuation at small scales (Table S3, Supporting Information).^[^ ^36^, ^37^ ^]^ Potential applications include assisting sperm with motility deficiencies in reaching the oocyte for in vivo assisted reproduction, targeted cancer therapy, management of infectious diseases, and tissue engineering, among others.^[^ ^38^, ^39^, ^40^, ^41^, ^42^, ^43^, ^44^, ^45^ ^]^ Nonetheless, concerns remain regarding residual materials left in the body, as their retrieval or degradation after therapy is often challenging.^[^ ^46^, ^47^ ^]^ Some studies have demonstrated combining catheters with untethered microrobots to enable their controlled release and retrieval. For instance, a flexible catheter equipped with a miniaturized electromagnet allowed precise release and localized actuation of microrobots deep within the body.^[^ ^48^ ^]^ Similarly, clinical endoscopes have been employed to deliver magnetic cell‐based robots for high‐precision stem cell therapy.^[^ ^49^ ^]^ Magnetically guided microcatheters have been reported to inject swarms of magnetic particles into the circulatory system.^[^ ^50^ ^]^ Another strategy utilizes multisegment catheters linked by biodegradable connectors, allowing the controlled release of untethered agents for precise interventions.^[^ ^51^ ^]^ And catheters incorporating permanent magnets have been used to retrieve nanoscale agents from the bloodstream.^[^ ^52^ ^]^ Despite the translational potential of these technologies, significant limitations persist, including the size constraints of electromagnet‐equipped catheters and endoscopes, restricted autonomous navigation, and difficulties in achieving scalable and reproducible fabrication.

**Figure 1:**
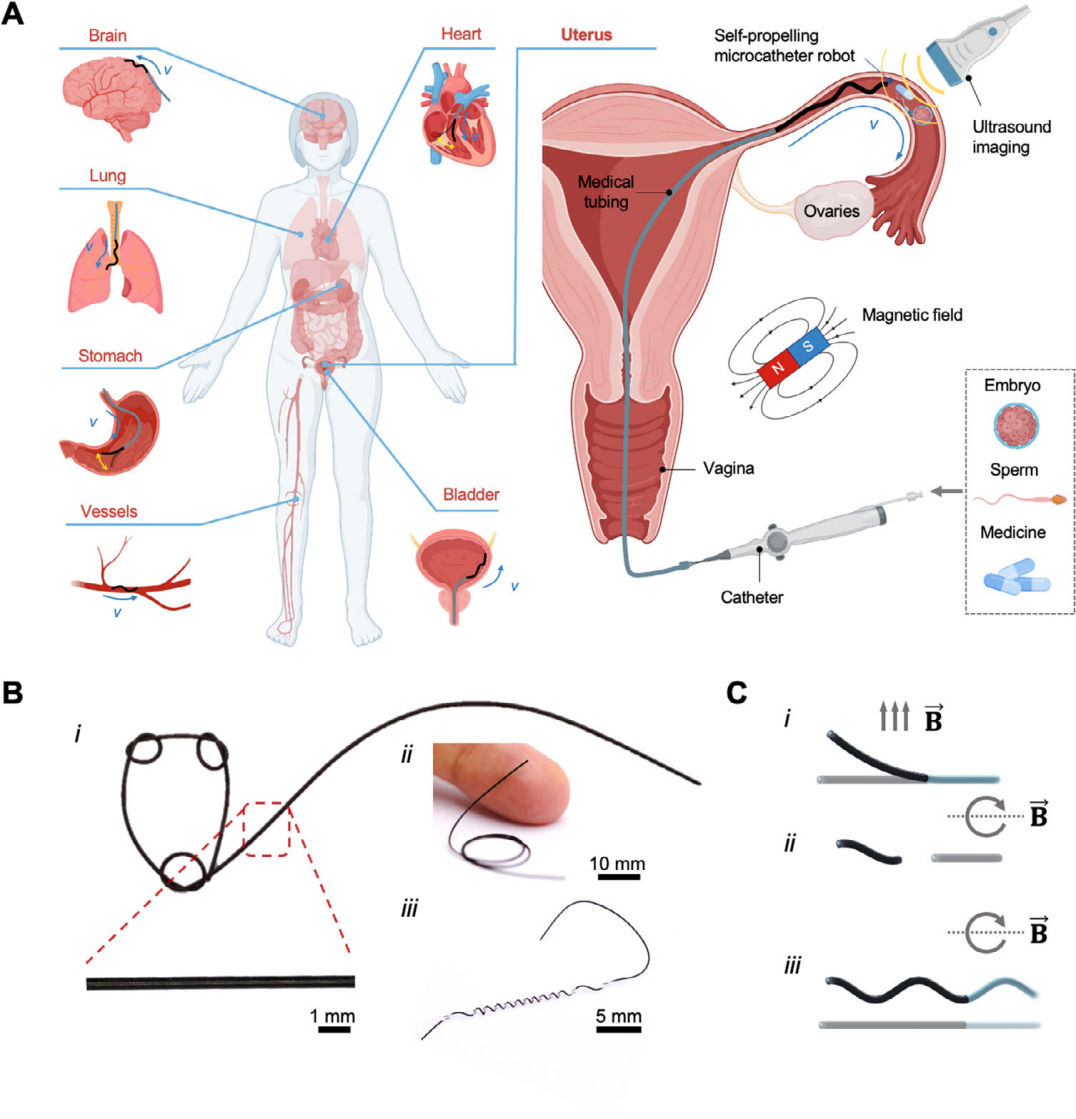
Schematic illustration of tubular tethered and untethered microrobots for diverse biomedical applications, highlighting their potential in precision reproductive medicine and targeted drug delivery. A) The microcatheter platform is designed to access hard‐to‐reach regions within the human body. B) The microcatheter, with an outer diameter of ≈400 µm, is soft and flexible, demonstrating high compliance as illustrated by its ability to wrap around a 1 mm‐diameter capillary. C) The platform supports multiple device configurations, including a guiding microcatheter (i), a tubular untethered microrobot (ii), and a hybrid microcatheter robot (iii).

Therefore, we suggest a fabrication approach for mass‐producing tubular magnetic microrobots, both tethered and untethered, that can be magnetized and operated across diverse microenvironments (**Figure** **1**A). These tubular structures feature lumen microchannels capable of housing drug‐loaded, stimuli‐responsive hydrogels or enabling the capture and release of cells and other therapeutic cargo. Inspired by the wave‐crawling propulsion observed in certain untethered microrobots, we introduce a hybrid motion strategy to enhance maneuverability: the distal portion of the microcatheter self‐propels while being gently pushed during insertion. The structures are fabricated using Joule heating with a wire template, allowing magnetization either during or after production (Figure 1; and Video S1, Supporting Information). We present three configurations: 1) a steerable guiding microcatheter with tunable stiffness; 2) a wave‐crawling tubular untethered microrobot (TubeBot) incorporating an enzyme‐digestible hydrogel, loaded with a model drug; and 3) a hybrid microcatheter robot combining distal wave‐crawling propulsion with linear insertion. The platform is validated by demonstrating navigation and cargo delivery across different models, including a soft tissue phantom, a 3D tortuous channel, a full‐scale human bronchial tree, 2D and 3D reproductive organ models, ex vivo mouse reproductive and respiratory organs, and an in vivo mouse model. Furthermore, we demonstrate targeted sperm delivery near the ampulla of the fallopian tube, for enabling artificial insemination, which is appealing in cases of male infertility, such as oligospermia (low sperm count) or asthenospermia (low sperm motility).^[^ ^53^, ^54^ ^]^ In this work, we also show the intrafallopian embryo transfer, relevant for cases of recurrent embryo implantation failure, which under in vivo conditions, can develop under natural, nutrient‐rich environment synchronized with endometrial preparation.^[^ ^47^, ^55^, ^56^ ^]^ Beyond reproductive medicine, we demonstrate that magnetic microcatheters can access distal lung regions for targeted therapy, aiming to enhance drug delivery to the alveoli or bronchioles, in contrast to conventional lung‐targeted therapies, which are typically administered systemically via intravenous injection, which is less precise and subject to hepatic metabolism. The methods and devices presented here, along with the supporting demonstrations, establish a solid foundation for biologically and clinically relevant studies. This paves the way for the platform's translational application in precision medicine, particularly in reproductive medicine, with potential for expansion into other biomedical fields.

## 2 Results and Discussion

### 2.1 Microrobots Fabricaton and Characterization

**Figure 2:**
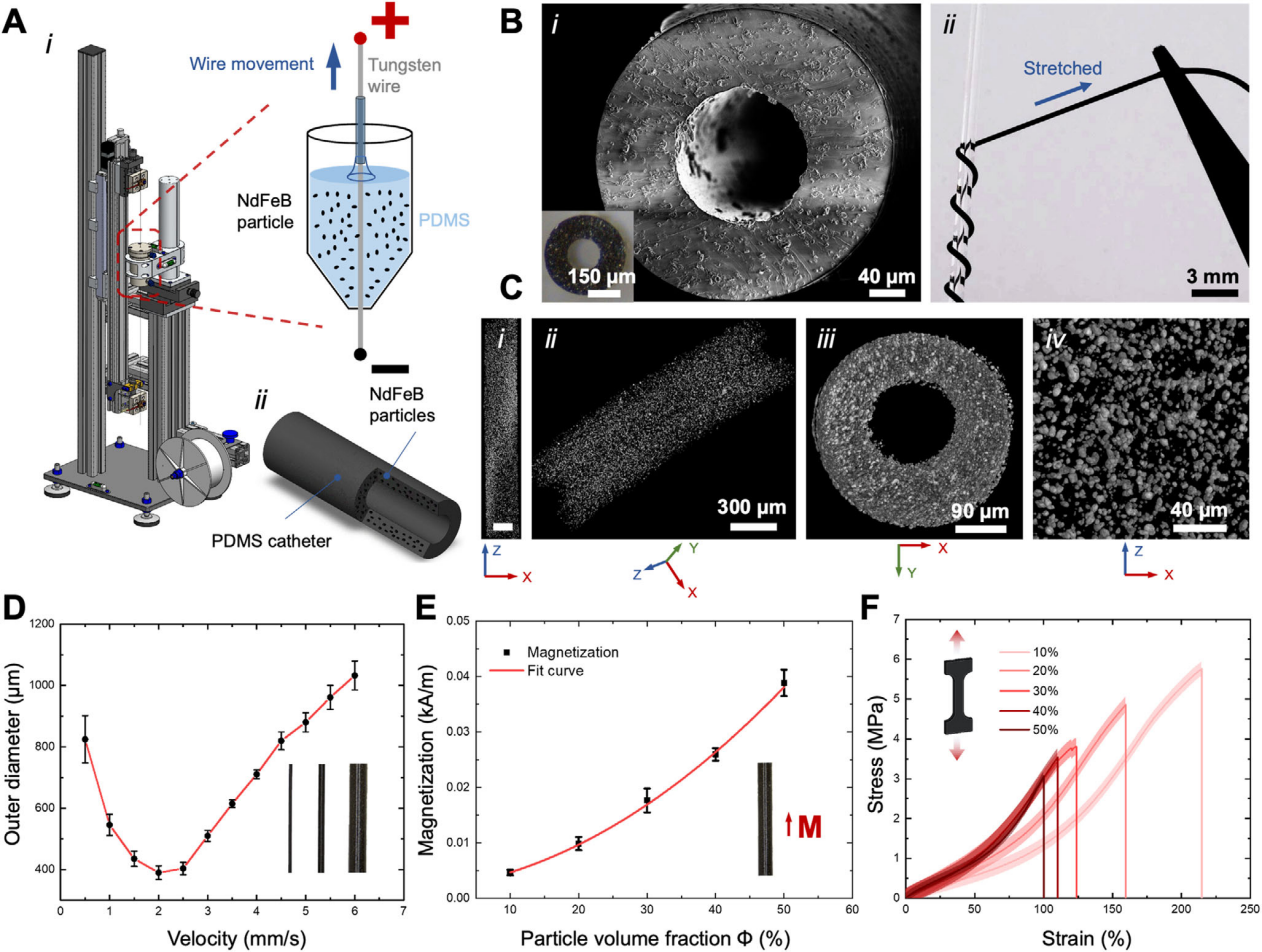
Fabrication and characterization of the magnetic microtubular platform. A) The fabrication setup based on the principle of Joule heating using a wire template, detailing the composite composition and the expected resulting geometry. B) SEM image of the microcatheter cross‐section (i) and a photograph of the microcatheter under tension (ii). C) MicroCT images show the distribution of NdFeB magnetic particles in the microcatheter at different magnifications, including longitudinal view (scale bar: 200 µm), (i) oblique view (ii), cross‐sectional view (iii), and local magnified view (iv). D) Control over the microcatheter diameter using the Joule heating principle, with variation achieved by adjusting the tungsten wire pulling speed. E) Magnetization (*M*) of the microcatheter as a function of the concentration of embedded magnetic particles (Φ). F) Stress–strain curves of ferromagnetic composites at different magnetic particle concentrations.

To enable rapid, low‐cost fabrication of magnetic microcatheters with micrometer‐scale diameters, we employed the Joule heating effect, to solidify a resin mixture through a continuous curing process (**Figure** **2**A(i); and Video S1, Supporting Information). The resin mixture consisted of polydimethylsiloxane (PDMS) resin (mixed at a 10:1 ratio) and uniformly dispersed NdFeB particles with a diameter of 5 µm. Ferromagnetic materials, such as NdFeB (neodymium‐iron‐boron) exhibit strong induced magnetization under an applied magnetic field. Unlike soft magnetic materials, which easily lose magnetization once the external field is removed, hard magnetic materials like NdFeB possess high coercivity, allowing them to retain significant remanent magnetization after the magnetic field is removed.^[^ ^57^ ^]^

The microcatheter fabrication setup is depicted in Figure S1, Supporting Information. The resin mixture was placed in a central container with tungsten wire running through its center, fixed at both ends to a displacement stage. A direct current was applied to the tungsten wire to generate heat, initiating the solidification of the resin mixture adhered to the wire. As the displacement stage gradually pulled the heated tungsten wire upward, the viscous resin coated the wire uniformly and solidified, forming the microcatheter. After manually removing the tungsten wire, a magnetic microcatheter was obtained, with its length determined by the tungsten wire's length. The structure of the fabricated microcatheter is shown in Figure 2A(ii), where NdFeB particles are uniformly embedded within the cured PDMS. Although PDMS was chosen for its thermal curing properties, we also successfully fabricated microtubular structures using Ecoflex, demonstrating the platform's compatibility with alternative materials exhibiting different elastic properties (Figure S2, Supporting Information).

Scanning electron microscopy (SEM) characterization of the microcatheter cross‐section (Figure 2B(i)) revealed a centrally located working channel, with a uniform and crack‐free wall structure. To further examine the internal distribution of NdFeB particles, X‐ray computed tomography was employed to obtain a 3D representation of the particle distribution (Figure 2C). Since the PDMS matrix signal was difficult to distinguish from the signal of the NdFeB, segmentation based on particle size, was performed to approximate the true 3D distribution of the particles (Figure S3, Supporting Information). The results indicated that the NdFeB particles were homogeneously distributed throughout the PDMS matrix with minimal overlap, confirming the uniformity of the microstructure.

The microcatheter's inner diameter was defined by the diameter of the selected tungsten wire used as a mold (with a diameter of 150 µm, chosen for demonstration purposes). Once the composite material has fully solidified, the speed of manual wire removal did not affect the final inner dimensions. Any minor variations observed are attributed primarily to slight thermal expansion of the wire during the heating process. However, we observed that the outer diameter was influenced by the pulling speed of the wire during heating, under constant power input to the tungsten wire (Figure 2D). As the pulling speed increased from 0 to ≈2 mm s^−1^, the outer diameter progressively decreased, reaching a minimum of ≈350–400 µm. However, at speeds beyond 2 mm s^−1^ up to 6 mm s^−1^, caused the diameter to rise again, reaching ≈1000 µm at the maximum speed. Thus, the fabricated microcatheters exhibited outer diameters ranging from 350 µm to 1 mm. At slow pulling speeds (0.5–2 mm s^−1^), the tungsten wire remains longer in the resin mixture, allowing more resin to cure on the surface and producing microcatheters with larger diameters (e.g., ≈800 µm at 0.5 mm s^−1^). As the pulling speed increases, the residence time in the resin decreases, and gravity reduces the amount of resin adhering to the wire, resulting in smaller diameters. However, at higher speeds (2–6 mm s^−1^), the outer diameter becomes primarily influenced by the resin's viscosity, which increases with pulling speed. The observed diameter increase at higher speeds is explained by hydrodynamic effects described by the Landau–Levich–Derjaguin theory (details found in the Supporting Information).

After production, we conducted a detailed characterization of the microcatheters, magnetic and mechanical properties. Microcatheters with five different mass fractions of NdFeB particles were fabricated and characterized (Figure 2E). The magnetization increased with particle concentration, but did not follow a strictly linear relationship due to the curing temperature affecting part of the magnetic properties of the NdFeB particles. Vibrating sample magnetometry (VSM) tests, conducted on ferromagnetic composite resin cured at different temperatures, revealed noticeable differences in remanent magnetization in the *M*(H) curves (Figure S4, Supporting Information). The microstructure of NdFeB particles near the tungsten wire is changed by the transient high temperature, which enhances the soft magnetic properties of NdFeB particles.^[^ ^58^, ^59^ ^]^ However, since the overall curing time was short, most NdFeB particles still retain their hard magnetic properties.

For the mechanical properties assessment, dog‐bone‐shaped specimens were fabricated for tensile testing using templates (Figure 2F).^[^ ^31^ ^]^ The results showed that the stress–strain behavior was consistent across different specimens, with the sample containing a 10% mass fraction of NdFeB exhibiting the highest tensile strain, while the 50% particle content sample showed the lowest tensile strain. Therefore, increasing the NdFeB particle content led to increased stiffness of the microcatheter, although even at 50% loading, the microcatheter retained 100% tensile deformation capacity. To gain deeper insight into how curing conditions influence the mechanical properties of the resulting ferromagnetic elastomer, atomic force microscopy (AFM) was employed. The Young's modulus was measured while systematically varying parameters such as curing time, curing temperature, and the elastomer‐to‐curing‐agent ratio (Figure S5, Supporting Information). The results indicate that increasing the curing temperature enhances the material's mechanical strength up to ≈120 °C. After that temperature, the material starts to degrade. Additionally, longer curing times and higher curing agent concentrations also impact the composite's mechanical strength, leading to increased hardness as these parameters are elevated.

### 2.2 Steerable Microcatheter

**Figure 3:**
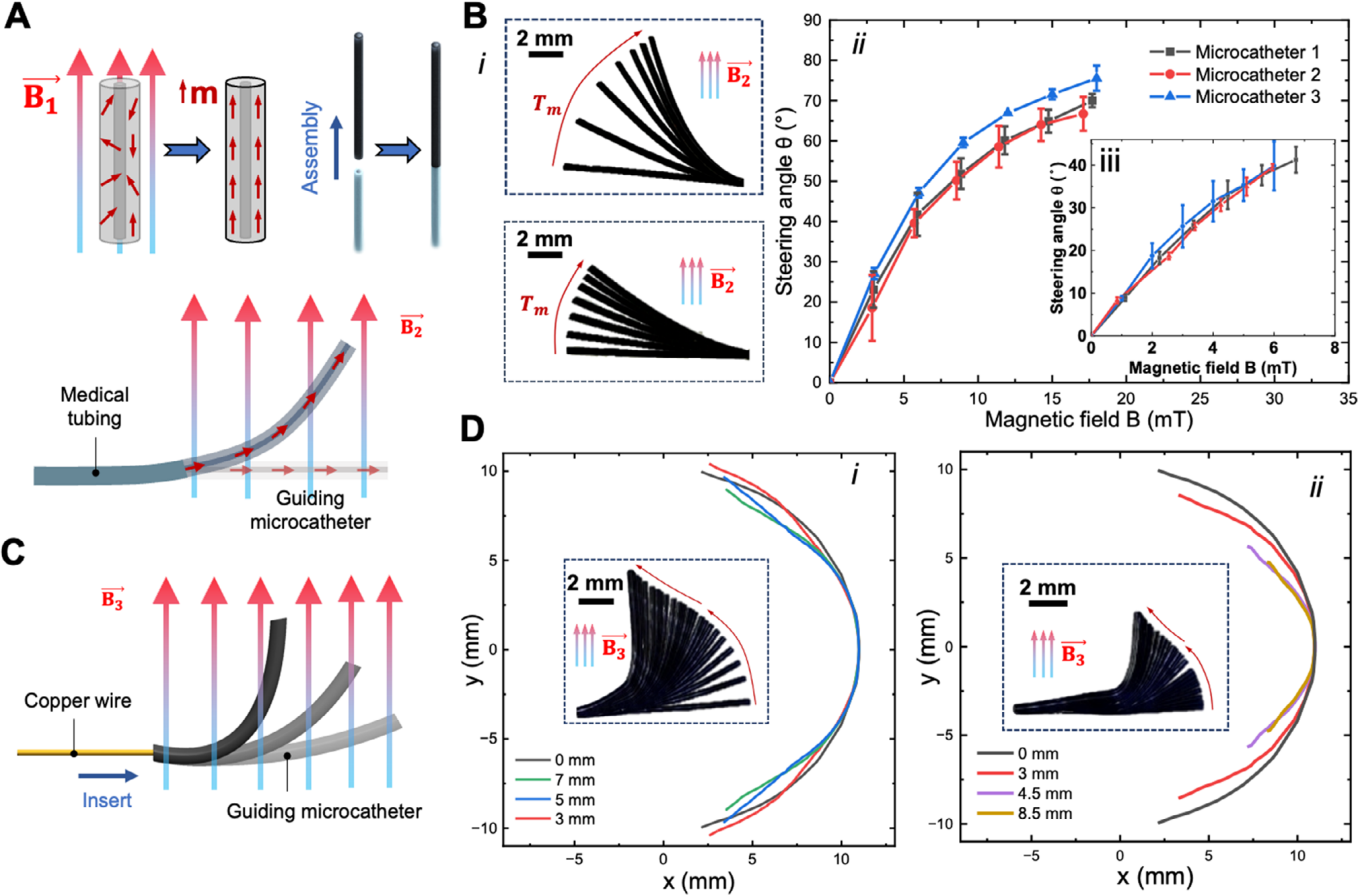
Characterization of a steerable microcatheter. A) Schematic diagram of a microcatheter with an internal magnetic moment m, magnetized under a uniform magnetic field $\vec{B_{1}}$ along the axial direction. The microcatether moves toward the direction of the magnetic field $\vec{B_{2}}$, applied perpendicularly to the body. B) The microcatheter achieves different angles (θ) of deflection due to the magnetic torque *T*_m_ resulted from the applied magnetic field $\vec{B_{2}}$. The magnetic field strength range is 0–18 mT (i), and the steering angle increases with field strength (ii). Notably, within the 0‐7 mT range, θ exhibits an approximately linear increase (iii). C) Schematic illustrating the insertion of a copper wire into the guiding microcatheter to locally tune its local stiffness. D) Motion performance of  the catheter‐Cu‐wire assembly under a magnetic field $\vec{B_{3}}$ (0–100 mT) for different copper wire diameters. Cu wire diameter tested: 50 µm (i) and 100 µm (ii).

The embedded NdFeB particles within the microcatheter were magnetized using a strong magnetic field (*B*_1_ = 3 T), resulting in a consistent magnetic moment (*m*) along the length of the microcatheter, thereby producing a steerable microcatheter (**Figure** **3**A). When an external uniform magnetic field ($\vec{B_{2}}$) is applied perpendicularly to the axis, the tip of the guiding microcatheter align with the field direction, resulting in the overall bending of the catheter. When coupled with medical tubing, the guiding microcatheter achieve effective steering and navigation under the influence of a uniform magnetic field.

To assess the microcatheter' steering capability, it was suspended in a magnetic field, demonstrating sufficient torque to overcome gravity (Figure 3B(i)). Further characterization involved measuring the steering angles of guiding microcatheters of identical diameter and length under different magnetic field strengths (Figure 3B(ii),(iii)). The results showed that the steering angle could reach up to 80° at 18 mT. Although the change in steering angle was not directly proportional to the applied field, the measurements were reproducible and confirmed the microcatheter's ability to achieve significant deflection at relatively low field strengths. Notably, at field strengths below 7 mT, the steering angle varied approximately linearly with the applied magnetic field, enabling precise control of small‐angle deflections (0°–40°).

The magnetic steering and selective navigation capabilities of the guiding microcatheter were further validated using a 2D maze (Figure S6 and Video S2, Supporting Information). The maze was designed with multiple branches (angles ranging from 0° to 50°) and a channel width of 1.5 mm. Magnetic steering was achieved using an external permanent magnet, which applied the magnetic torque needed for turning, while manual pushing was used to propel the medical tubing forward.

Altering the operational workspace requires changing the length of the guiding microcatheter in free space, which imposes stringent requirements on both operations and in vivo space.^[^ ^60^ ^]^ To address this, materials were inserted or infused into the microcatheter's central channel to locally alter the overall device's stiffness, thereby enabling the tip to reach various positions within the body cavity without changing the length of the guiding microcatheter. Copper wires were chosen for insertion into the working channel to form stiffness difference (Figure 3C). By adjusting the length of the inserted copper wire, various tip positions were achieved under the influence of an external magnetic field ($\vec{B_{3}}$, 0–100 mT).

Copper wires with diameters of 50 µm (Figure 3D(i)) and 100 µm (Figure 3D(ii)) were inserted into the catheter, and the deflection of the guiding microcatheter tip was measured for different insertion lengths. The tip achieved a deflection of up to 90°, and its trajectory was recorded throughout the deflection process. When a magnetic field was applied with copper wire inserted, the tip trajectory differed from the one without wire (0 mm insertion). Increasing the length of the copper wire reduced the tip's trajectory radius, which was more pronounced when using the 100 µm wire (e.g., the 3 mm insertion case for both 50 and 100 µm wires). This indicates that steering and workspace adjustments can be achieved in more intricated channels.

In addition, to more clearly demonstrate the operational workspace of the guiding microcatheter in a uniform magnetic field due to the difference in stiffness, copper wires of different lengths with a diameter of 150 µm were inserted into the working channel (Figure S7, Supporting Information). By using the built‐in wire with higher stiffness, the effective flexible length of the guiding microcatheter was adjusted, enabling large‐angle navigation within confined spaces. The structural integrity of the microcatheters after repeated wire insertions and retractions was confirmed through optical microscopy and fluid leakage tests (Figure S8, Supporting Information), demonstrating their robustness and suitability for repeated use in such procedures. While copper wire was employed to illustrate the principle of stiffness modulation, future iterations will utilize clinically proven materials, such as nitinol, which provide superior biocompatibility and tunable mechanical properties for navigating complex anatomical pathways.

### 2.3 Untethered Microrobot (TubeBot)

**Figure 4:**
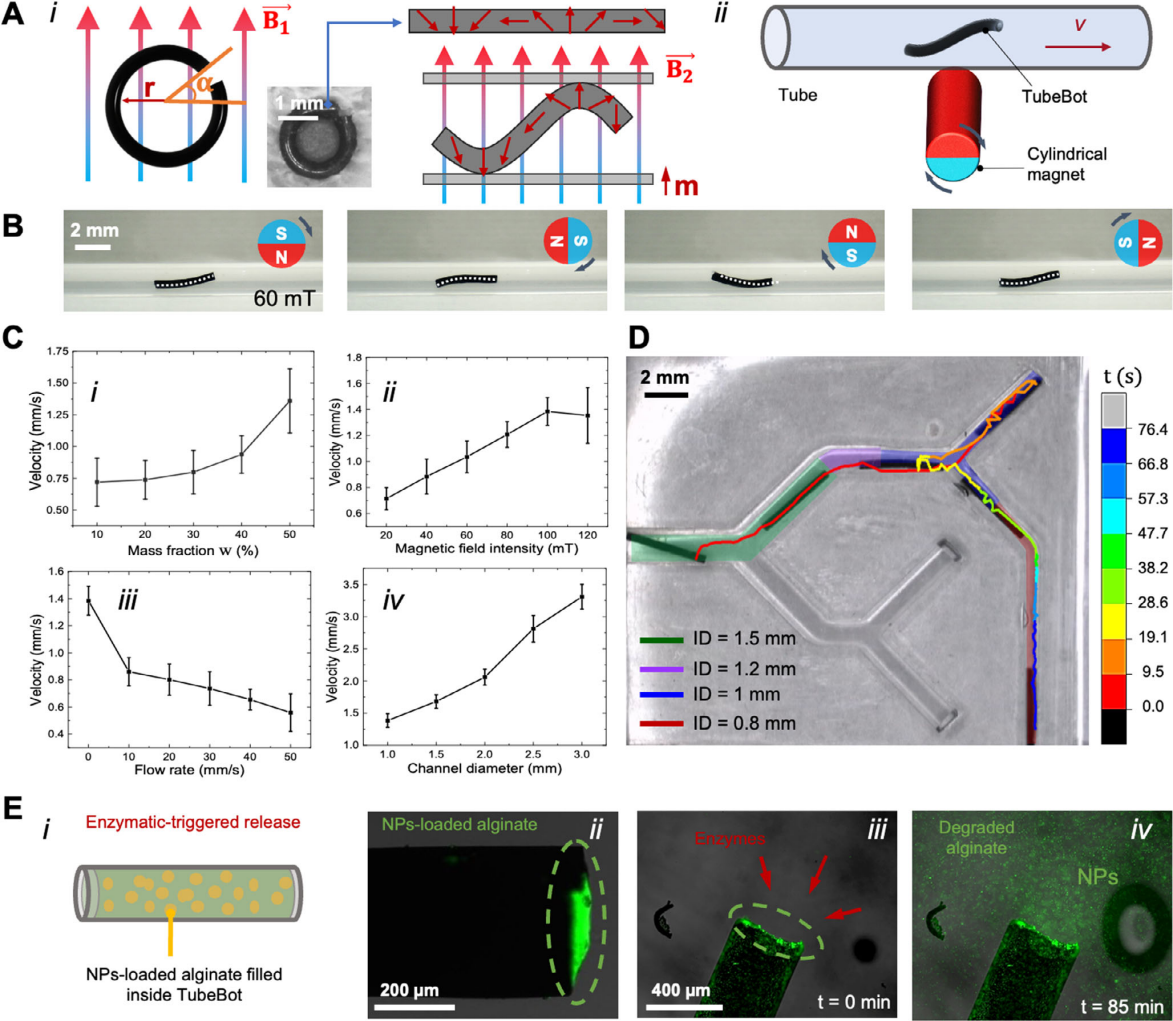
Characterization of the untethered tubular microrobot (TubeBot). A) TubeBot is fixed within a mold (radius = 1 mm) and magnetized using a uniform magnetic field $\vec{B_{1}}$. After magnetization, its relaxed (free) state is straight. Upon application of an upward uniform magnetic field $\vec{B_{2}}$, TubeBot adopts a wavy configuration within the confined space (i). Schematic illustration of the experimental setup used to evaluate TubeBot's performance (ii). B) Sequential postures of TubeBot under different orientations of a rotating magnetic field inside, a silicone phantom tube (inner diameter = 1 mm). C) Parameters influencing the crawling speed of TubeBot: i) increasing magnetic particle content enhances propulsion, ii) higher magnetic field amplitude results in faster motion, iii) in the presence of fluid flow, crawling speed varies linearly with the flow rate, and iv) in silicone tubes of increasing diameters, crawling speed increases. D) TubeBot navigating and crawling through a channel with variable diameter. Overlaid images show the microrobot's sequential positions along the channel. E) Schematic representation of an alginate gel plug for cargo transport within TubeBot (i). Fluorescence microscopy image of TubeBot loaded with alginate containing fluorescence nanoparticles (ii). Time‐lapse images of TubeBot motion through the channel toward a target location while carrying the alginate‐basedcargo (iii). At *t* = 0 min, the alginate is fully loaded. At *t* = 85 min, enzymatic degradation of alginate results in the release of the fluorescent nanoparticles.

A wireless, untethered microrobot, designed as a segment of the produced tubular microcatheter, was also demonstrated. This design combines the benefits of catheter‐like geometry with the flexibility of wireless microrobotics. Compared to other untethered magnetic microrobots, these are easier to produce at scale and can be magnetized in various patterns along their length. Their lumen allows for the loading of diverse cargo, including drug‐embedded stimuli‐responsive hydrogels, as well as cell‐based materials. However, it is important to note that other techniques can provide other advantages. For example, two‐photon lithography allows the fabrication of intricate 3D structures, but requires cleanroom facilities and is unsuitable for large‐scale production.^[^ ^61^ ^]^ Likewise, If the need is for micro/nanorobots of very small size and in large quantities, chemical synthesis may be the preferred method.^[^ ^62^ ^]^ Additionally, template‐based methods like electrodeposition and micromolding are particularly suited for producing smaller microtubular structures but require the use of conductive or ionic material.^[^ ^63^ ^]^ In contrast, our proposed method focuses on continuous production of tubular soft structures, which, through an integrated cutting step, can lead to scalable production of tubular microrobots in varying lengths and diameters. For the demonstration, a microcatheter segment was cut to 4 mm in length and fixed in a cylindrical mold with a radius of 0.5 mm for magnetization. A uniform magnetic field (*B*_1_ = 3 T) was applied perpendicularly through the mold and microcatheter, ensuring an initial phase shift of 45° for the magnetization (**Figure** **4**A(i)) resulting in a wave‐crawling tubular microrobot.^[^ ^64^ ^]^

To characterize the programmed magnetization of the microrobots, a magneto‐optical Kerr effect (MOKE) microscope was used (Figure S9, Supporting Information). The alternating bright and dark areas with two nodes along the microcatheter, confirmed that the internal magnetic distribution was consistent with the design, demonstrating the feasibility of magnetization programming. Without an external field, TubeBot remained a straight horizontal line. When a uniform magnetic field ($\vec{B_{2}}$) was applied perpendicular to the TubeBot's body axis, the embedded NdFeB particles trended to align with the external field, generating magnetic torque and causing deformation. When placed in a confined space and subjected to a rotating magnetic field, the shape of TubeBot becomes sinusoidal, enabling crawling propulsion.

To evaluate TubeBot's propulsion within a vessel‐like environment, a 1 mm diameter silicon tube, filled with phosphate‐buffered saline (PBS) solution was used, and a cylindrical permanent magnet generated the rotating magnetic field (Figure 4A(ii)). Upon applying the rotating magnetic field, TubeBot motion over one rotation cycle was recorded for further analysis (Figure 4A(iii),B). Due to the body length not being significantly greater than the diameter, TubeBot did not exhibit a complete sinusoidal shape (the white dashed line represents TubeBot's posture).^[^ ^64^ ^]^ Testing the crawling direction of TubeBot revealed that it moved in the same direction as the magnetic field rotation (Figure S10, Supporting Information).

To optimize TubeBot's crawling performance, we studied various TubeBot designs under different applied magnetic fields and navigation environments (Figure 4C). TubeBot's crawling speed was evaluated by using different mass fractions of magnetic particles (*w*) under a magnetic field of 60 mT, 5 Hz. As shown in Figure 4C(i), the crawling speed increased significantly as *w* increased. For mass fractions between 10 and 30%, the speed remained around 0.75 mm s^−1^ with little variation. However, at *w* greater than 30% up to 50%, the speed increased markedly, reaching ≈1.5 mm s^−1^. Higher magnetic particle content significantly increased TubeBot's stiffness, resulting in reduced deformation under the magnetic field and unstable crawling speed, thus limiting further increases in particle content.

TubeBot's crawling speed under different rotating magnetic field strengths (Figure 4C(ii)) showed an approximately linear increase, ranging from about 0.7 to 1.5 mm s^−1^, as the amplitude of the field increased from 20 to 100 mT, with the magnetic field rotation frequency held at 5 Hz. However, a slight decrease in crawling speed was observed when the field strength reached 120 mT. This reduction could be attributed to increased deformation of TubeBot at higher magnetic field strengths, which led to changes in its crawling posture, thereby affecting the crawling performance. Additionally, at higher magnetic field strengths, the attraction of the rotating permanent magnet to TubeBot increased, further impeding its forward motion and introducing greater variability.

The crawling speed of TubeBot under different magnetic field rotation frequencies (Figure S11, Supporting Information) exhibited a linear growth trend as the frequency increased from 2 to 16 Hz, with a magnetic field strength of 100 mT. The crawling speed increased from ≈0.25 to 3.25 mm s^−1^, and the experimental data showed good agreement with the linear fit, indicating a direct correlation between rotation frequency and crawling speed. Based on the exploration of the effect of external magnetic parameters on TubeBot's crawling performance and for the convenience of data collection, subsequent characterization of TubeBot's motion, including factors, such as magnetization angle, resistance to fluid, varying tube diameters, magnetization angle, magnetization diameter, and robot length, was conducted using a rotating magnetic field of 5 Hz and 100 mT, unless otherwise stated.

The crawling speed of TubeBot is strongly influenced by its magnetization angle, α, which is defined as the angle between one end of the TubeBot and the direction of the applied magnetic field (Figure 4A(i)). The magnetization angle affects the initial phase shift of the internal magnetization distribution, thereby altering the TubeBot's crawling posture and speed. As shown in Figure S11 (Supporting Information), the crawling speed reached its peak when the initial magnetization angle was set to 45°. This suggests that a 45° magnetization angle provides an internal magnetization distribution that most closely resembles a sinusoidal wave, resulting in more efficient crawling motion.

Moreover, the ability of TubeBot to move within fluid‐filled environments was assessed by testing its performance in a silicone tube with various flow rates of PBS (Figure 4C(iii)). TubeBot crawled against the direction of the PBS flow, and the flow velocity was controlled using a micropump. Initially, TubeBot's crawling speed decreased rapidly, followed by a more gradual decline as the PBS flow rate increased. At a flow rate of 50 mm s^−1^, TubeBot maintained a net forward speed of 0.6 mm s^−1^. For reference, venous blood flow in human leg veins has a peak velocity of 200–300 mm s^−1^ and a mean velocity of 20–40 mm s^−1^.^[^ ^65^ ^]^ Thus, TubeBot demonstrated the potential to crawl against venous blood flow in vessels.

In addition to straight pathways, in vivo channels are typically nonuniform in diameter. Therefore, TubeBot's performance in varying channel diameters was evaluated (Figure 4C(iv)). As the channel diameter increased from 1.0 to 3.0 mm, TubeBot's crawling speed showed an approximately linear increase, from about 1.35 to 3.25 mm s^−1^. This result indicates that TubeBot achieved higher crawling efficiency in larger‐diameter channels. Moreover, we investigated the influence of different magnetization diameters and TubeBot lengths on crawling speed (Figure S11, Supporting Information). The results indicated that variations in magnetization diameter led to asymmetric magnetization within TubeBot of the same length, resulting in different left‐right crawling speeds. The relationship between TubeBot length and crawling speed was approximately linear, with longer TubeBots exhibiting decreased speed. Interestingly, we identified a critical trade‐off between the TubeBot's length (with the same magnetization profile) and its maneuverability in tortuous environments (Figures S12 and S13, Supporting Information). While longer TubeBots can generate stronger propulsive forces, their increased rigidity can hinder effective navigation through successive sharp bends. In contrast, our experiments consistently show that shorter TubeBots exhibit superior performance in navigating complex, multiturn channels. These findings underscore the importance of optimizing robot length for specific anatomical requirements, a flexibility made possible by our fabrication platform. TubeBots for various testing requirements were magnetized following different schemes, as illustrated in Figure S14 (Supporting Information). Since the TubeBot's locomotion depends on the interaction between its undulating body and the channel wall, its performance is inherently influenced by the surface properties of its operating environment. Although not systematically investigated in this study, variations in surface roughness and lubricity, such as those found in mucus‐lined airways versus vascular endothelium, are likely to affect crawling behavior. Highly lubricious surfaces may reduce the friction necessary for effective propulsion, leading to slippage and decreased velocity. In contrast, higher‐friction surfaces could improve traction but may also increase resistance to movement, necessitating greater driving forces, as previously reported.^[^ ^64^, ^66^ ^]^

Based on previous testing, the optimal TubeBot parameters were determined as follows: 50% mass fraction of NdFeB particles, 4 mm length, 45° initial magnetization angle, and a magnetization diameter of 1 mm. To further validate TubeBot's performance in a vessel‐like branched structure, experiments were conducted to observe TubeBot's motion through channels of varying inner diameters, and its trajectory was recorded over time (Figure 4D; and Figure S15 and Video S3, Supporting Information). Velocity profiles were calculated based on these trajectories (Figure S16, Supporting Information). The results indicated that TubeBot's speed increased with channel diameter in the four different inner diameter channels (0.8, 1, 1.2, and 1.5 mm). TubeBot exhibited the highest speed in the 1.5 mm channel, while its speed was relatively lower in the 0.8 mm channel. Notably, TubeBot required more time to navigate branch points, largely due to manual adjustments of the rotating magnetic field to guide its path. Time‐lapse images showed TubeBot's trajectory at various time points, indicating stable performance even in channels with diameters close to TubeBot's outer diameter. When changing the path, the TubeBot moves forward and backward by changing the direction of the magnetic field, which shows that the TubeBot is highly maneuverable even in a variable‐path channel.

TubeBot's adaptability to varying channel diameters was further tested using a stepwise tapered channel model constructed from silicone tubes of different diameters (1, 2, and 3 mm) (Figure S17 and Video S3, Supporting Information). TubeBot successfully adjusted to changes in diameter and crossed intermediate steps regardless of whether it was transitioning from smaller to larger diameters or vice versa.

To evaluate TubeBot's cargo‐loading and releasing capability, TubeBot loaded with alginate hydrogel was used to demonstrate its movement through a maze phantom and release at the target point (Figure 4E; and Figure S18, Supporting Information). Alginate loaded with fluorescent nanoparticles was used to visualize the process, and TubeBot successfully carried and maintained the integrity of the material (Figure 4E(i),(ii)). Alginate, a hydrogel that can be compressed in aqueous solution after cross‐linking. TubeBot's crawling is primarily driven by body deformation, and the loaded alginate did not hinder TubeBot's deformation under the magnetic field (Figure S19A, Supporting Information). Crawling tests in a sloping tube further demonstrated TubeBot's ability to move in complex environments while carrying the cargo (Figure S19B, Supporting Information). In the maze phantom, TubeBot navigated through different pathways (with branch angles θ_1_, θ_2_, and θ_3_) and successfully reached the target point *a* (Figure S18; and Video S3, Supporting Information). TubeBot efficiently navigated through branches of varying angles while loaded, demonstrating its load‐carrying capacity and efficient movement in complex channels. After reaching the target point, TubeBot released the alginate in a 37.5 °C environment over 24 h, verifying its release capability (Figure 4E(iii,iv); and Video S3, Supporting Information). As time progressed, alginate was gradually released under enzymatic action, with the distribution of fluorescent nanoparticle at 85 min demonstrating the release process (Figure 4E(iv). This experiment confirmed TubeBot's potential as a drug delivery platform.

To assess the effects of TubeBot's crawling on cell activity, experiments were conducted to evaluate its movement over cell layer. TubeBot was tested within a bovine oviduct epithelial cells (BOECs) model (Figure S20, Supporting Information). TubeBot crawled smoothly over the BOECs layer with continuous reciprocating movement, without causing any interference or adhesion to the cells. Fluorescence staining was used to evaluate cell viability, and images showed that the BOECs layer remained intact after multiple passes by TubeBot, with no evidence of widespread cell damage or significant reduction in cell viability. These findings indicate that TubeBot's movement over the BOECs layer was gentle enough to avoid physical damage, validating its safety in biological environments. Details of the experiments are in the “TubeBot testing in BOECs model” section of the Supporting Information.

### 2.4 The Microcatheter Robot

**Figure 5:**
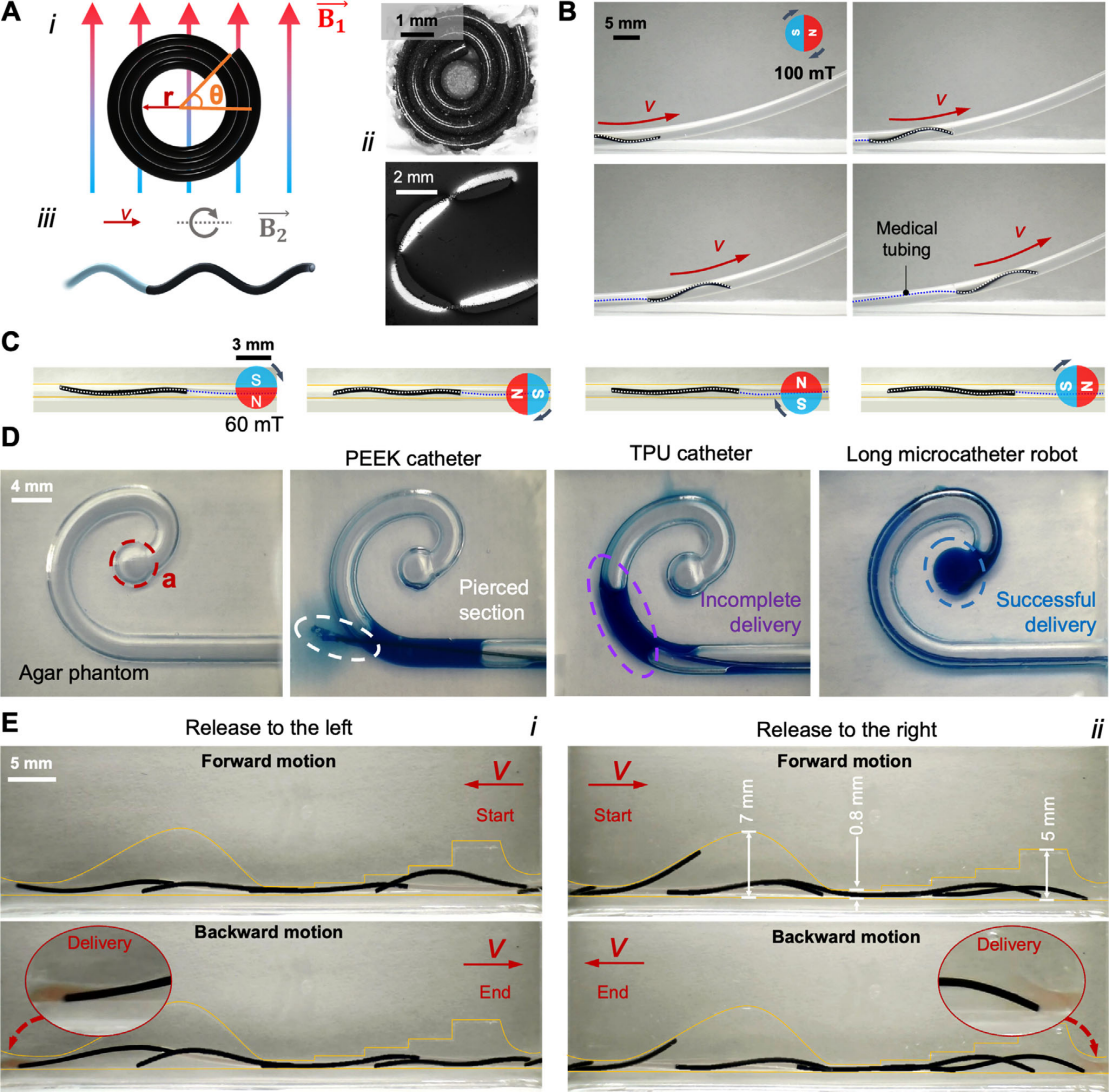
Microcatheter robot optimization and function demonstration. A) The microcatheter robot is fixed in a mold (*r* = 1 mm) and magnetized using a uniform magnetic field $\vec{B_{1}}$ (i). Visualization of the magnetization distribution using MOKE microscopy (ii).The microcatheter robot attached to medical tubing is driven by a rotating magnetic field $\vec{B_{2}}$ (iii) B) The microcatheter robot crawls upward through a sloped silicone tube (*ID* = 2 mm) without external pulling. C) Postures of the microcatheter robot at various angles of the rotating magnetic field in a silicone tube (*ID* = 1 mm). D) Advancement of different medical tubes inside an agar phantom. i) Overview of the complete agar phantom. ii) The PEEK medical tubing (*d* = 0.38 mm) passes through the channel within the agar phantom. iii) The TPU catheter (*d* = 0.64 mm) becomes lodged midway along the channel. iv) The microcatheter robot successfully crawls to target point *a* and transfers liquid. E) The microcatheter robot demonstrates robustness by crawling and advancing within a complex channel (yellow outline). i) The microcatheter robot crawls forward from right to left, then backward from left to right, effectively transporting liquid (red). ii) When connected to medical tubing, the microcatheter robot crawls forward from left to right and backward from right to left within a complex channel.

To equip the microcatheter with self‐propulsion capabilities for effective navigation in complex tissue, a design similar to TubeBot was developed, but with increased length. The resulting micro catheter robot features a magnetization radius (r) of 0.5 mm, wrapped in three helical turns, with a total length of 15 mm (**Figure** **5**A(i)). The innermost loop has a magnetization diameter of 1 mm, which gradually increases for the subsequent loops, but both ends of the magnetized catheter remain aligned on the same axis, with an initial magnetization angle set to 45°. The internal magnetic moment distribution after magnetization, shown in Figure 5A(ii), confirms the diameter variation.

Upon connection to medical tubing, the microcatheter robot achieved terminal active propulsion under a rotating magnetic field, propelling the medical tubing forward (Figure 5A(iii)). The microcatheter robot was placed in an inclined channel with an inner diameter of 2 mm and driven by a rotating magnetic field of 100 mT. The results demonstrated that the microcatheter robot was able to overcome gravity and successfully dragged the medical tubing upward without external assistance (Figure 5B), confirming its capability of distal active propulsion.

The microcatheter robot was subjected to different magnetic fields in a channel with a diameter of 1 mm, resulting in deformation over a magnetic field rotation cycle (Figure 5C). The white dashed line indicates the posture of the magnetic catheter component of the microcatheter robot, which took on a complete sinusoidal shape, generating continuous propulsive forces. The blue dashed line indicates the posture of the medical tubing. The capability of the microcatheter robot to deliver material in simulated biological environments was evaluated using an agar phantom with compliance comparable to that of human soft tissue.^[^ ^60^ ^]^ The performance of different catheters in delivering to target point *a* was compared (Figure 5D). The polyetheretherketone (PEEK) catheter failed to navigate through the agar phantom as its stiffness prevented it from bending to follow the path, resulting in puncture (Figure 5D(ii)). The thermoplastic polyurethane (TPU) catheter failed to reach the target point and got stuck in the middle of the phantom because, without terminal deflection, the static friction between the catheter and the phantom impeded its advancement (Figure 5D(iii)). In contrast, the microcatheter robot demonstrated superior navigation capabilities in the agar phantom, effectively crawling to target point and successfully delivering fluid without damaging the phantom (Figure 5D(iv)).

To evaluate the robustness of the microcatheter robot's motion capabilities, it was placed in a complex channel to perform both forward and reverse crawling to deliver fluids (Figure 5E; and Video S4, Supporting Information). The channel outline was highlighted with yellow lines, and the upper portion of the channel featured an asymmetric structure, as detailed in Figure S21 (Supporting Information). During the leftward delivery experiment (Figure 5E(i)), the microcatheter robot crawled forward from right to left, passing through a descending step and an arched section, with the narrowest part having a channel height of 0.8 mm. During the return process, the microcatheter robot successfully delivered red fluid along the edge of the channel, before moving in reverse from left to right.

In the rightward delivery experiment (Figure 5E(ii)), the microcatheter robot navigated through the arched section first, followed by the step section, delivering fluid and then reversing from right to left. The experimental results indicate that regardless of the direction of movement in the complex channel, the microcatheter robot was able to adapt to changes in the channel structure and achieve effective fluid delivery. The robot's robustness in navigating complex channels demonstrates its high adaptability and delivery stability, further highlighting its potential advantages for practical clinical applications.

### 2.5 Microcatheter Robot Navigation in Anatomical Phantom Models

**Figure 6:**
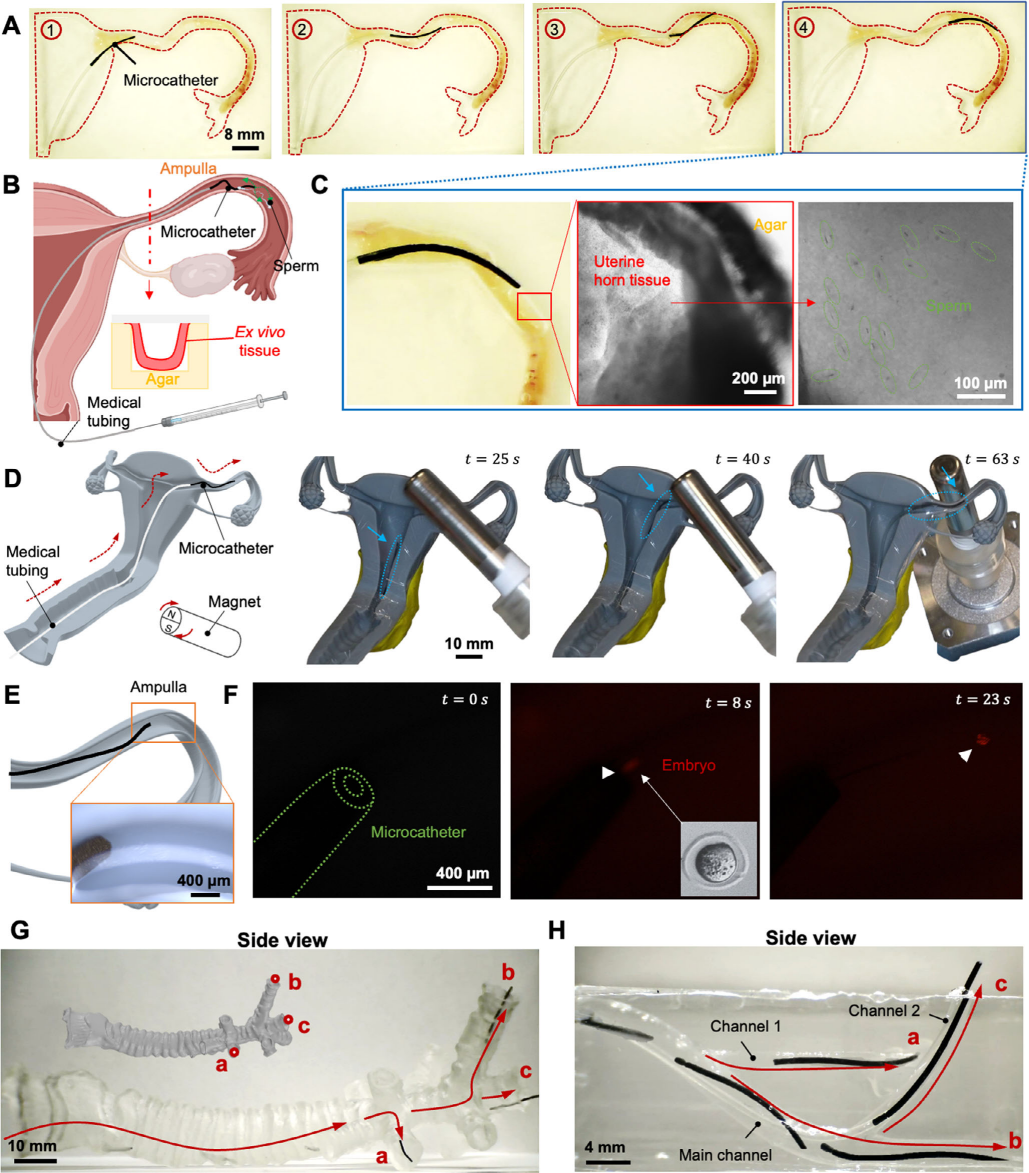
Microcatheter robot navigation in 3D Anatomical Phantom Models. A–C) Sperm delivery in a tissue‐lined reproductive system model: the robot navigates a tortuous channel lined with ex vivo rat uterine tissue (A), illustrated in cross‐section image (B), showing the delivery of a sperm sample at the fertilisation site of the Fallopian tube, (C). D–F) Zygote delivery in a scaled‐down (2.5×) human reproductive phantom: the robot traverses from the vaginal orifice through the uterus to the fallopian tube ampulla under magnetic actuation (D), assisted by a rotating permanent magnet to the ampulla site of the fallopian tube (E), releasing a fluorescently labeled zygote via a microfluidic pump over 20 s(F). G) Full‐scale 3D bronchial tree navigation: side view of the robot progressing through the left and right bronchial trees, reaching sequential target points a–d over 184 s, driven by a rotating magnetic field and manual pulling. H) Navigation in a branched 3D channel (main channel 3 mm, side channels 1.5 mm): the robot moves through side channels and main pathways, reaching multiple target points (a–c) over 130 s under magnetic guidance and manual assistance.

Here, we evaluated the microcatheter robot in various anatomical models to demonstrate its capability for navigating complex anatomical structures (**Figure** **6**; and Video S5, Supporting Information). Thus, we first developed a high‐fidelity pseudo‐3D phantom featuring a section of the reproductive tract lined with rat uterine tissue (Figure 6A–C). The robot successfully navigated this complex pathway and delivered sperm cells, demonstrating its potential for delicate interventions within intricate biological conduits. This approach offers a promising strategy for artificial insemination, allowing sperm release closer to the fertilization site due to the catheter's gentle motion and compact size (Figure 6C; and Video S5, Supporting Information). However, there remains a potential risk of damaging the delicate ciliated epithelium during wave‐crawling motion in vivo.^[^ ^67^ ^]^ To maximize applicability while minimizing disruption to ciliary function and avoiding polyspermy, only a small number of in vitro–capacitated sperm at low concentrations should be released near, but not directly at the fertilization site. Ciliary cells are unevenly distributed along the fallopian tube: in the isthmus (the narrowest segment near the uterus), ciliary abundance is below 40%, and the lumen diameter ranges from 1 to 5 mm, larger than our microcatheter. At the fertilization site, between the ampulla and infundibulum, ciliary abundance exceeds 80%. By combining a precise insertion mechanism with a controllable microfluidic pump, it is possible to release a low number of sperm just before the ampulla, preserving ciliary integrity. This approach is particularly relevant for cases of oligospermia or asthenospermia, where sperm count or motility is limited.^[^ ^53^, ^54^ ^]^ Nevertheless, further studies are needed to fully evaluate the impact on ciliary function and subsequent embryo transport in future in vivo applications.

Additionally, we Suggest an alternative procedure in which early‐stage embryos are reintroduced into the fallopian tube to develop under physiological conditions before transfer to the uterus. This concept builds on the historically effective but invasive zygote intrafallopian transfer (ZIFT), which relies on laparoscopic procedures and has been limited by procedural variability, operator dependence, and risks, such as ectopic pregnancy.^[^ ^13^, ^18^ ^]^ ZIFT was traditionally considered for patients with repeated embryo implantation failure or when conventional assisted reproduction techniques (using conventional intrauterine embryo transfer) had been unsuccessful. Its relevance is particularly pronounced for women over 35, when fertility begins to decline exponentially.^[^ ^68^ ^]^ To further demonstrate the navigation and delivery capabilities of the robot within female reproductive anatomy, a zygote was successfully delivered near the ampulla of the fallopian tube in a scaled‐down (2.5×) human reproductive 3D phantom generated based on CT images of an actual reproductive tract (Figure 6D; and Video S5, Supporting Information).^[^ ^69^ ^]^ The microcatheter was navigated from the cervix to the fallopian tube, and a single embryo, modeled using a murine embryo, with dimensions closer to the one of human embryos, was released, with its presence confirmed via fluorescence microscopy due to the difficulty of observing it under brightfield (BF) mode (Figure 6E,F). It is also worth noting that the use of retrievable magnetic microtools, as an alternative to untethered microrobots (previously reported by our group for assisted reproduction applications), prioritizes clinical feasibility and safety by preventing the retention of residual materials. This work builds on our earlier strain‐engineered microcatheter systems for controlled release of embryo‐sized particles with integrated sensors and tracking, which lacked active actuation and sufficient flexibility.^[^ ^70^ ^]^ The current design incorporates magnetic actuation to overcome these limitations, with the long‐term goal of developing a multifunctional, retrievable system combining sensing and actuation. We believe this strategy offers significant advantages over standard IVF for difficult‐to‐treat patient groups, establishing a strong rationale for its clinical translation as a complementary tool to enhance fertility outcomes.

Another underexplored medical scenario is navigation through the bronchial tree. This procedure is performed using a flexible bronchoscope, which is inserted into the trachea and gradually advanced into the deeper branches of the bronchial tree, reaching at most the smaller airways of the lung.^[^ ^71^ ^]^ During bronchoscopic procedures, a guiding catheter often serves as an anchor to deploy and stabilize other catheters or tools, while a flexible catheter is advanced toward the target area.^[^ ^72^ ^]^ However, these procedures remain relatively invasive due to the size of the bronchoscope, with even the smallest models having an outer diameter of ≈3 mm, and have been primarily demonstrated in animal studies or experimental therapies. The most commonly adopted route for delivering therapies to the lung is systemic administration, either via intravenous or oral drugs, or via inhalation.^[^ ^73^ ^]^ Given the complexity of the bronchial tree, additional guidewires or instruments may be required, increasing both procedural difficulty and strain on the patient's airways. The process demands considerable skill and often requires multiple adjustments, particularly in cases of anatomical abnormalities or narrow airways. Repeated insertions and equipment changes not only prolong the procedure but also increase the risk of damage to the bronchial mucosa, bleeding, and other complications.^[^ ^74^ ^]^ Minimizing the use of catheters and guidewires can therefore improve surgical efficiency and safety, reducing both potential risks and overall procedural time.

Therefore, in this proof‐of‐concept demonstration, we tested the tubular microcatheter using a complete 3D bronchial tree model, reconstructed from CT images of a real patient's organ, to assess its ability to navigate anatomically accurate and complex pathways (Figure 6G; and Figure S22A and Video S5, Supporting Information). Both the side view and top view (Figure 6G; and Figure S22A and Video S5, Supporting Information) illustrate the microcatheter's robust movement through the bronchial model. After entering the main bronchus, the robot successfully crawled through bends ranging from 10° to 60°, covering a distance of over 316 mm in 160 s.

Finally, the microcatheter was tested in a simplified 3D channel featuring multiple side branches (Figure 6H; and Figure S22B, Supporting Information). The microcatheter entered the main channel at *t* = 0 s, advanced into channel 1 (*d* = 1.5 mm), and traversed bends ranging from 10° to 40° in 3D space within 100 s, even when operated by a user with limited experience. This demonstrates the device's capability to navigate complex branching pathways within a defined timeframe, supporting potential clinical applications. A comparison of the microcatheter's performance in side branches using manual pushing versus Distal propulsion (Figure S23, Supporting Information) revealed that manual pushing combined with magnetic steering could result in jamming within narrow or sharply curved channels. In contrast, terminal crawling under magnetic navigation provided greater robustness, allowing the microcatheter to successfully traverse challenging sections.

### 2.6 Microcatheter Actuation in Ex Vivo Organs under Ultrasound Guidance

**Figure 7:**
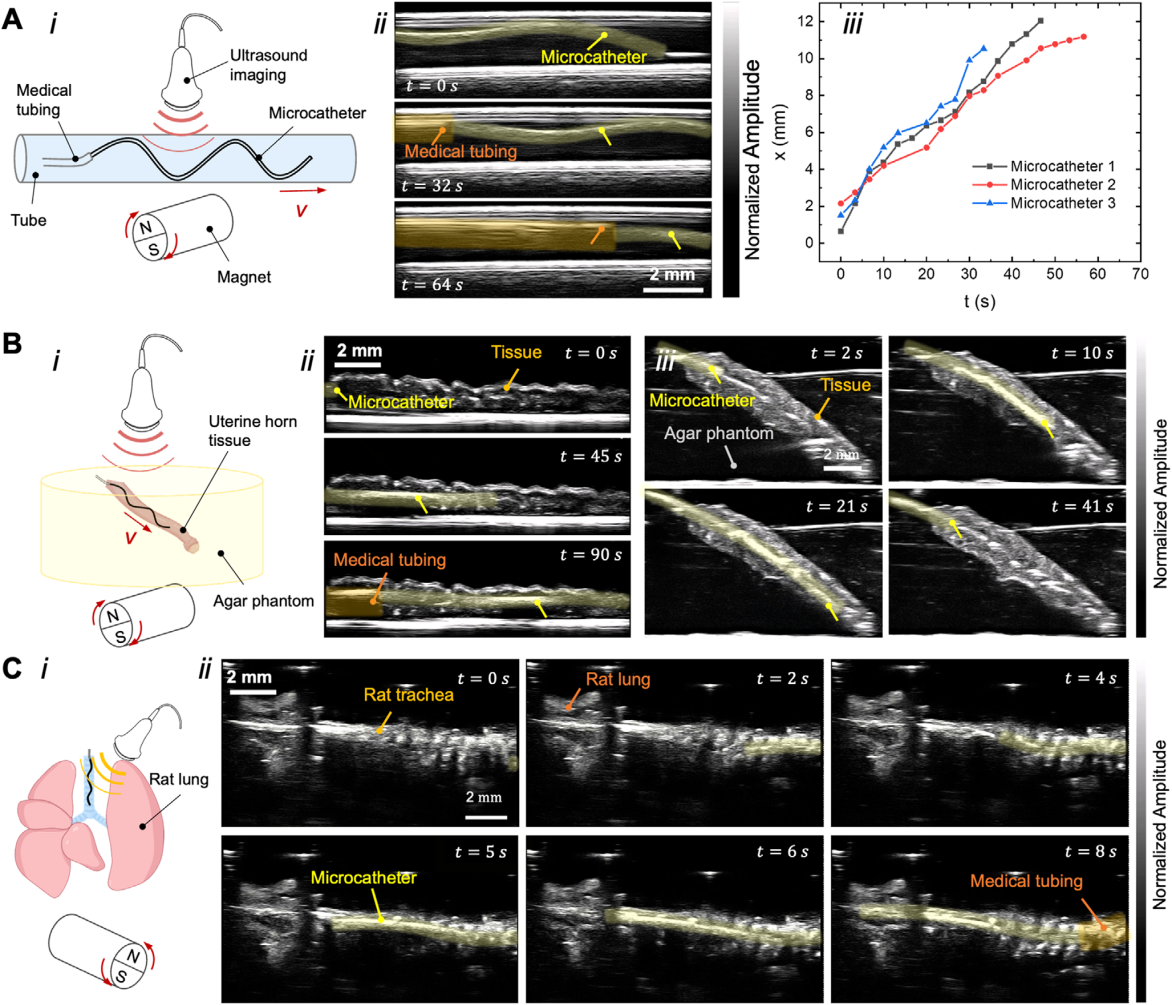
Ex vivo microcatheter robot actuation under ultrasound guidance. The microcatheter robot is imaged crawling through A) a tube phantom while pulling, The medical tubing B) uterine tissue embedded in an agar phantom, and C) Ultrasound Imaging provides real‐time visualization of the microcatheter robot's position and motion within a rat lung via the trachea. In the tube phantom, the robot's speed under a 100 mT/7 Hz magnetic field is quantified. Across all models, the robot successfully navigates complex pathways, demonstrating precise magnetic actuation and strong potential for minimally invasive interventions.

To enable real‐time visualization of the microcatheter robot, an ultrasound (US) imaging system was used to monitor its movement within an ex vivo phantom model, mouse uterine tissue, pig trachea, and rat lung, thereby evaluating its navigation capability and stability in complex biological environments (**Figure** **7**; and Figure S24 and Video S6, Supporting Information). Figure 7A shows the microcatheter robot's movement in the tubing model for imaging parameter's optimization. As depicted in Figure 7A(i), the experimental setup included a rotating magnetic field generated by a permanent magnet coupled to a step motor to drive the robot's motion, with an ultrasound probe in contact with the tube using ultrasound gel to ensure signal transmission. The actual settings are shown in Figure S25 (Supporting Information). The US imaging (Figure 7A(ii)) captured the microcatheter robot (highlighted in yellow) as it dragged a medical tubing (highlighted in brown) under magnetic actuation. Speed data presented in Figure 7A(iii) demonstrated consistent movement speeds across different microcatheters under identical magnetic field conditions, indicating that the microcatheter robot maintained stable crawling velocity and effectively completed the task of crawling and dragging the medical tubing within the channel phantom setup.

Further experiments evaluated the microcatheter robot's movement within ex vivo mouse uterine tissue (Figure 7B; and Figure S24, Supporting Information). Figure 7B(ii) displays US imaging of a microcatheter robot crawling within mouse uterine tissue on an acrylic surface, where significant signal reflection from the bottom interferes with the observation of the microcatheter robot. The use of agar effectively reduced signal reflection from the acrylic base, resulting in clearer US imaging of the microcatheter robot (Figure S24A, Supporting Information). Figure 7B(iii) further demonstrates the robot's movement within uterine tissue embedded in an agar phantom with acoustic properties similar to those of biological soft tissue.^[^ ^75^ ^]^ The preparation of uterine horn tissue embedded in agar allowed the formation of an environment resembling a 3D in vivo structure after agar solidification. Under US imaging guidance, the robot successfully crawled along the uterine horn, reaching the base of the phantom, and ultimately Crawling backward to exit the phantom without any significant hindrance movement.

**Figure 8:**
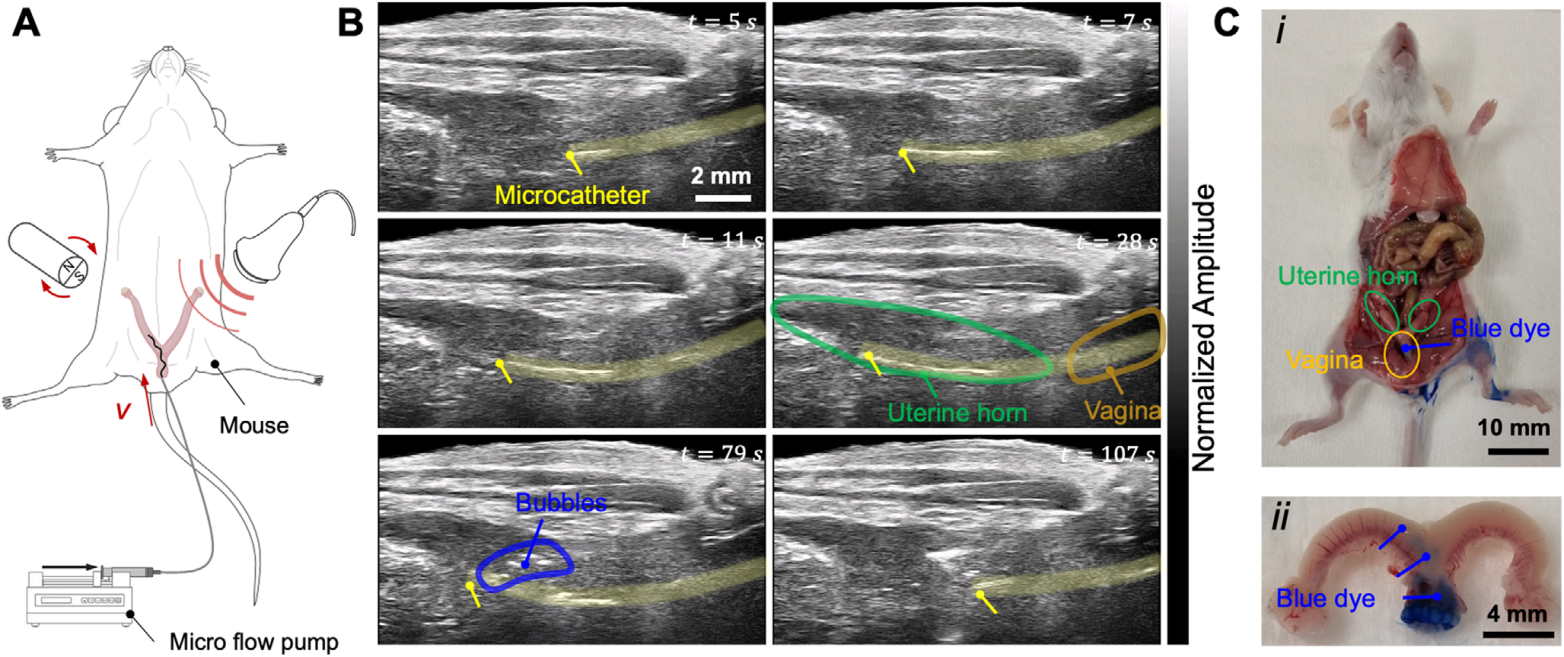
Ultrasound‐guided microcatheter robot actuation in a living mouse uterus. A) Schematic of ultrasound imaging showing the microcatheter robot crawling in the uterus and delivering a dyed solution. B) The microcatheter robot navigates the uterus via the vagina and delivers a dyed solution injected through its lumen. C) Postmortem verification: i) uterine staining, and ii) blue dye marking the delivery site on the left uterine horn.

To further demonstrate its versatility for other anatomical targets, the movement and deflection of the microcatheter robot in rat lung and pig trachea ex vivo were also successfully verified under US imaging guidance (**Figure** **8**C; and Figure S23, Supporting Information). As shown in Figure 7C(i), a permanent magnet was placed under the isolated rat lung, and the microcatheter robot entered the lung tissue through the trachea. Figure 7C(ii) shows the robot crawling through the rat trachea into the lung tissue, and the rotating magnetic field was used to guide the robot into the right lung. It was verified by changing the US imaging direction to display the US image of the vertical plane of the lung (Figure S24B, Supporting Information). These experimental results validated that under real‐time US imaging guidance, the microcatheter robot could reach target points within both the channel and ex vivo biological tissue, exhibiting consistent speed and stability across different environments.

### 2.7 In Vivo Microcatheter Robot Performance

To demonstrate the clinical applicability of this technology, an in vivo feasibility study was conducted on a mouse model. Although the female mouse reproductive system differs from the human in size and anatomy, it is commonly used as a preliminary model because its fundamental physiological functions are comparable.^[^ ^76^ ^]^ The microcatheter robot was employed to navigate and deliver fluid, mimicking semen or therapeutic agents, within the mouse reproductive tract, with its movement and positioning monitored in real‐time via US imaging (Figure 8). The experimental setup (Figure 8A) involved inserting the microcatheter robot through the vagina and cervix, after which it gradually navigated and crawled toward the uterine horn under a rotating magnetic field. The microcatheter robot was connected to a medical tubing and a syringe containing a blue dyed solution (Figure S26, Supporting Information). Once positioned, fluid delivery to the target site was carried out using a microfluidic pump. US imaging illustrated the robot's movement within the mouse reproductive system (Figure 8B). At *t* = 5 s, the robot entered the mouse's vagina (yellow‐marked region), and by *t* = 28 s, it had successfully navigated to the uterine horn (green‐marked region). The US images demonstrated the robot's movement from the vagina through to the uterine horn. At *t* = 79 s, the blue dyed solution was pumped through the microcatheter robot's tip and released into the uterine horn. The movement and dispersion of bubbles within the blue‐marked dye region indicated smooth, unobstructed dye delivery. After the experiment, euthanasia was performed, and following organ dissection, confirming the successful delivery of the dye to the uterine horn (Figure 8C(i),(ii)). The results demonstrated that the microcatheter robot could effectively navigate and deliver fluids within a complex in vivo reproductive environment, opening new ways for minimally invasive drug delivery or more effective in vivo assisted insemination.

## 3 Conclusion and Outlook

This research introduces a high‐yield fabrication method to produce tubular magnetic microrobots, tethered and untethered, enabling rapid, cost‐effective customization for a wide range of biomedical applications. Compared to other approaches, the continuous Joule heating method offers superior scalability and production efficiency (Table S4, Supporting Information), allowing reproducible fabrication of soft magnetic microtubular devices of virtually unlimited length, compatible with various thermo‐curable materials (Figure S2, Supporting Information). Moreover, these tubular devices can be programmed with specific magnetization patterns to create robotic platforms with configurable propulsion and navigation capabilities, optimized for complex anatomical pathways.

Unlike traditional rigid guidewires that require frequent manual adjustments, our soft‐material‐based microcatheters incorporate embedded wires for tunable stiffness, reducing tissue trauma while allowing precise modulation of their workspace. The wave‐crawling propulsion mechanism provides the microcatheter tip with self‐propulsion, enhancing robustness, and enabling effective navigation in tortuous channels and organ environments. Distal propulsion minimizes perforation risk by preventing proximal forces from being transmitted directly to the tip, while the soft‐tipped design further improves safety. Minor vibrations induced by wave‐crawling are absorbed by the catheter's softness, minimizing tissue damage, as demonstrated by BOECs‐coated phantom tests (Figure S20, Supporting Information). To compare the forces exerted during the deployment of our wave‐crawling microcatheter with those of a conventional push‐based TPU catheter, we conducted experiments on a cell‐lined fluidic channel. Cell viability was assessed using both fluorescence microscopy and a quantitative XTT assay, which measures cell proliferation and metabolism. The push‐based approach resulted in a visible scratch, indicating substantial cell removal and damage, whereas the wave‐crawling motion preserved the overall integrity of the cell layer, with only minor reductions in cell viability (Figure S29, Supporting Information). In contrast to existing end‐actuated catheters and state‐of‐the‐art research and commercial steerable microcatheters, our system offers significant advantages in size, fabrication flexibility, propulsion efficiency, and the integration of functional micrometer‐scale working channels (Tables S5 and S6, Supporting Information). Moreover, by combining proximal manual advancement with distal active crawling, navigation through complex anatomical segments is achieved in ≈40% less time than with distal crawling alone, as shown in maze navigationexperiments (Figures S27 and S28, Supporting Information). Future integration of automated linear actuators could further enhance efficiency and manipulation accuracy.

In addition to the two tethered microcatheter configurations (the guiding microcatheter and the microrobot catheters), we also introduce an untethered variant, here termed TubeBot, which further expands the platform's potential for cooperative, multiscale therapeutic interventions. These untethered units enable precise access to the final centimeters of the target site (Figure 4).^[^ ^51^ ^]^ TubeBot has demonstrated the ability to deliver drugs into confined anatomical regions, using embedded hydrogels which digest in presence of local proteases.

Similar advances by Lukas et al. showcased a customizable magnetic soft microrobot fabricated via a microfluidic extrusion approach.^[^ ^51^ ^]^ Upon reaching the target site, this device decomposes into a microrobot swarm to achieve highly localized therapeutic delivery. This example highlights the broader potential of flexible continuous robots and microrobots to operate cooperatively within a shared platform, enhancing one another's functional efficiency.

Within our platform, TubeBot and the tethered microcatheter robot are designed as complementary components, with TubeBot seamlessly integrable into the microcatheter system. Looking ahead, this integration offers a pathway toward more efficient and targeted delivery mechanisms, enabling precise, multiscale interventions.

Other important considerations include the use of surface coatings to further enhance biocompatibility and optimize friction during longer procedures. Embedding magnetic particles within polymers provides a balance between flexibility and magnetic strength, while increasing particle content or employing softer matrices can mitigate magnetic field attenuation. At present, magnetic navigation relies on permanent magnets (≈100 mT). A critical consideration for clinical translation is the rapid decay of magnetic field strength with distance. Overcoming this challenge requires a multipronged strategy that combines device optimization with advances in actuation systems. A key advantage of our platform is the ability to enhance the microcatheter's responsiveness, thereby lowering the required field strength. This can be achieved by increasing the concentration of embedded magnetic particles to boost the intrinsic magnetic moment, or by fabricating the device from softer polymer matrices (e.g., Ecoflex, Figure S2, Supporting Information) to reduce the mechanical stiffness and thus the torque required for wave‐crawling locomotion. On the other side, the actuation platform involves either mounting high‐strength permanent magnets on robotic arms, programmed to track the device to the desired location.^[^ ^77^, ^78^, ^79^ ^]^ Or by using advanced electromagnetic coil arrays, which can dynamically focus the magnetic field at clinically relevant depths.^[^ ^35^ ^]^ In both cases, the applied magnetic field can be a magnetic field gradient, a rotating magnetic field, or a combination of both. In fact, the clinical feasibility of integrating large‐scale actuation systems into the operating room is well established, as demonstrated by existing platforms like the Stereotaxis Niobe and Genesis RMN systems, which have been used for complex cardiac electrophysiology procedures, and which are commercially available.^[^ ^30^ ^]^ The success of these systems underscores that the engineering and logistical challenges inherent to more robust magnetic guidance can be surmounted when significant clinical advantages justify their implementation.

Compared to traditional systems, the absence of haptic feedback remains a major challenge. Surgeons rely on tactile cues from conventional guidewires to navigate and minimize tissue damage. While some devices incorporate pressure sensors, the forces generated by flexible microcatheters are typically too small and dissipate through deformation and friction, yielding little useful data during surgery.^[^ ^80^, ^81^ ^]^ Future approaches may overcome this by estimating contact stress from real‐time imaging (e.g., US) and integrating force sensors at the catheter tip to enhance navigation accuracy.

Although promising, comprehensive in vivo testing and large‐scale trials are essential for regulatory approval and clinical deployment, including evaluation of ciliary integrity in the case of application in the reproductive tract, tissue compatibility, vibration effects, and navigation accuracy. Future integration of force sensors and advanced imaging modalities, such as pseudo‐Doppler ultrasound, could improve real‐time feedback, further enhancing safety and efficacy in clinical scenarios.^[^ ^70^, ^80^, ^81^, ^82^, ^83^ ^]^

We demonstrated that the magnetic materials embedded within the microcatheter also act as effective ultrasound contrast agents, enabling real‐time actuation and tracking in both ex vivo organs and living mice. To further enhance in vivo tracking accuracy, on‐board electronic sensors can be integrated for magnetic localization, as shown in our previous smart microcatheter work in which magnetoresistance sensors in presence of an oscillatory magnetic field were employed to determine the position of the microcatheter under scattering tissues^[^ ^70^ ^]^ and by other groups employing magneto‐oscillatory sensors.^[^ ^84^ ^]^ While the incorporation of additional sensors can enhance safety and maneuverability, it also introduces challenges related to catheter size and the available lumen space.

Overall, the key are its rapidity, cost‐effectiveness, and suitability for laboratory settings, thanks to its compact size and user‐friendly interface. By adjusting the tungsten wire diameter during fabrication, microcatheters of various sizes and working channel diameters can be easily produced. The platform also enables the use of different materials, and, within a single process, it is possible to create multimaterial catheters. While our continuous fabrication method demonstrates excellent yield and customization at a research scale, additional engineering and market analysis would be necessary to evaluate its economic viability for large‐scale industrial production. From a medical translation perspective, our research primarily focuses on assisted reproduction, where microcatheter robots can precisely navigate the uterine or fallopian tubes to deliver sperm or early‐stage embryos with minimal tissue disruption. This platform allows targeted delivery near fertilization sites, potentially improving outcomes in cases of oligospermia, asthenospermia, or repeated embryo implantation failure. Beyond reproductive applications, microcatheter robots also show promise in other organ systems, such as the lungs, where they can traverse bronchial trees for targeted drug delivery or localized interventions, offering minimally invasive alternatives to rigid bronchoscopes or conventional catheters. While these findings are promising, comprehensive in vivo testing and large‐scale clinical trials are essential for regulatory approval and widespread clinical adoption. These steps will require extensive risk assessments and validation to ensure the safety and effectiveness of the technology in real‐world settings.

## 4 Experimental Section

### Preparation of Magnetic Microcatheter

NdFeB microparticles (MQFP‐B‐10215‐089, Magnequench) with an average particle diameter of 5 µm were uniformly mixed into the PDMS resin base (Sylgard 184, Dow Corning) to prepare a ferromagnetic composite resin. NdFeB microparticles (10–50 weight % (wt%)) were mixed into the PDMS silicone base using a paddle stirrer (AR‐100, Thinky) at 2000 rpm and stirred for 2 h. After the resin solution mixed with NdFeB microparticles was cooled to room temperature, the curing agent was added in a weight ratio of silicone base to curing agent of 10:1 and mixed thoroughly. Next, the resin was degassed under vacuum to remove bubbles inside the resin. The magnetic microcatheter was prepared by Joule heating of a tungsten wire, and the preparation device is shown in Figure S1 (Supporting Information).^[^ ^85^ ^]^ The ferromagnetic composite resin was slowly transferred into the container, and a tungsten wire (7440‐33‐7, Goodfellow) with a diameter of 0.15 mm was passed through the container and fixed at both ends of the electric displacement platform. A constant current of 2.3 A was applied to the tungsten wire, and at the same time, the electric displacement platform (X‐LSM200A‐E03, Zaber) was used to move the tungsten wire upward at a constant speed. The Joule heat generated by the tungsten wire solidified the ferromagnetic composite resin on the tungsten wire. After the tungsten wire was removed, the microcatheter on the tungsten wire was manually and mechanically peeled off.

### Fabrication of Phantoms

The models were prepared using two different techniques. The first method was to print different resins using a 3D stereolithography printer (Mars 4, ELEGOO) to obtain a single flat or 3D model. The channels and molds were drawn using CAD software and 3D printed using resin (Build, Siraya Tech) and transparent resin (ABS‐like Resin, ELEGOO). The complex channels on the flat surface were combined with a cover plate of Polymethyl Methacrylate (PMMA). The molds of 3D structures were molded using PDMS (10:1 mass ratio) to prepare different types of channels and bonded to glass slides to obtain ideal closed phantoms. The bonding process was to plasma treat the PDMS model and a clean glass slide for 1 min at a power of 99 watts, then adhere the PDMS model to the glass slide and bake it on a hot plate at 120 °C for 20 min. In addition, the tracheal tree was 3D printed in one go using transparent resin. For the agar phantom, agar (9002‐18‐0, Sigma‐Aldrich) and deionized water were mixed at a mass ratio of 3%, heated to 100 °C, and manually stirred to dissolve. Wait until the agar solution is clear, cool to 40 °C, and pour into the 3D printed mold. When cooled to room temperature, remove the agar phantom. The second method is to prepare the 3D channel phantom using the template method. The mold model was designed using CAD software, PMMA was cut accordingly to obtain mold parts, and finally hot glue (Bigstren) was used to get the mold. Put silicone tubes (Adtech polymer engineering Ltd) with different outer diameters (1.5, 3 mm) into the mold, and use super glue (Stanger) to connect the silicone tubes. After standing for 1 h, slowly pour PDMS (10:1 mass ratio) into the mold and vacuum exhaust. Place the mold containing PDMS on a hot plate at 65 °C overnight to ensure that the PDMS is completely polymerized. After removing the glue between the mold parts, disassemble the mold to expose the PDMS model containing the silicone tube inside. Because the contact area between the silicone tubes is small and the silicone tubes are elastic, the silicone tubes inside the PDMS can be manually pulled out, and it is ensured that there is no solidified super glue blocking the internal channel.

### Magnetic Characterization

The magnetic moment density of ferromagnetic composite microcatheters with different NdFeB particle concentrations was measured using a vibrating sample magnetometer (DMS 1660, ADE Technologies). The samples were prepared by cutting microcatheters with an outer diameter of 300 µm into 5 mm lengths and fixing them into the sample holder of the magnetometer. The external magnetic field was applied up to 3 T and cycled twice. When the applied external magnetic field was zero, the residual magnetic moment of the sample was measured and then divided by the sample volume to obtain the magnetization or magnetic moment density of the sample.

### Mechanical Testing

The dog‐bone‐shaped ferromagnetic composites with different NdFeB particle concentrations were prepared by 3D printing molds. The specimens used for tensile testing had a test width of 4 mm and a gauge length of 17 mm. The specimens were tensile tested on a mechanical testing machine (Z005, Zwick/Roell) using a 2.5 KN load cell at a test rate of 50 mm min^−1^.

### Radiography Characterization

To assess the distribution of the NdFeB magnetic particles in the microcatheter X‐Ray computed tomography (XCT) (Phoenix nanotom m, General Electric) has been conducted. Scans were performed at 80 kV and 120 µA with a voxel size of 1 µm. Samples were prepared by cutting microcatheters with 300 µm outer diameter into roughly 5 mm lengths and clueing them on sample holder for XCT. To illustrate the tomographic reconstruction in different magnifications and views, including longitudinal, oblique, cross‐sectional, and local magnified view, the Software VGStudioMax 2023.4 has been used.

### Magnetization of Microcatheter Robots

After the microcatheter embedded with NdFeB particles is peeled off from the tungsten wire, the microcatheter is personalized for different functional applications. All the demonstrated magnetic microcatheter prototypes are uniformly magnetized by a pulsed magnetic field (3T) generated by a pulsed magnetizer (IM2525MAC, Magnet‐Physik) so that the NdFeB particles are magnetically saturated. The guiding microcatheter is magnetized axially, and the microcatheter is fixed in the vertical groove of the 3D‐printed mold during magnetization. The magnetization of the TubeBot adopts a programmed magnetization curve that approximates a sinusoidal magnetization curve. The microcatheter is fixed to the mold at 45° with the starting end and the magnetic field direction, close to a cylinder with a diameter of 1 mm, and ensures that the entire microcatheter is in one plane. The magnetization of the microcatheter robot is similar to that of TubeBot. The starting end of the microcatheter robot is an axial magnetized end with a length of 1 mm as the navigation end, and then the remaining microcatheter part is programmed to be magnetized using a sinusoidal magnetization curve. It is also necessary to ensure that the entire microcatheter is in one plane during magnetization.

### Magneto‐Optical Kerr Effect Characterization

A magneto‐optical indicator film with perpendicular anisotropy (PMOIF) is employed to visualize the magnetization distribution within a microcatheter. The core of this PMOIF is a transparent magnetic garnet film, which is deposited on a transparent gadolinium‐gallium‐garnet (GGG) substrate and coated with a mirror layer (Figure S9, Supporting Information). In the absence of an external field, the magnetic garnet film naturally forms a ground state of alternating, up‐and‐down magnetized band domains. When positioned on the surface of a magnetic sample, the stray field originating from the sample's poles causes modifications in the perpendicular garnet band domain, changing the “density” of up‐ or ‐down‐domains. By using a magneto‐optical wide‐field Kerr microscope configured for polar sensitivity under low‐resolution settings—where the individual garnet domains are not visible—a polar Faraday contrast is produced in the reflection polarization microscope.^[^ ^86^ ^]^ This contrast pattern mirrors the pole arrangement within the cylindrical microcatheter revealing the magnetization distribution in it.^[^ ^87^ ^]^

### Microcatheter Robot's Cargo Loading and Release

A microcatheter with a length of 4 mm was programmed to magnetize with a sinusoidal magnetization curve, and the microcatheter was treated with plasma (99 watts) for 1 min. Alginate (9005‐38‐3, Sigma‐Aldrich) was mixed into deionized (DI) water at a mass ratio of 5:100 to prepare an alginate solution. The alginate solution was diluted to 0.5% (mass ratio) and vortex‐mixed with fluorescent nanoparticles (26 988, Duke Scientific Corporation) at a volume ratio of 30:1. One end of the microcatheter was connected to a capillary (Hilgenberg GmbH) with a tip outer diameter of ≈140 µm, and the alginate solution containing fluorescent nanoparticles (particle size of 200 nm) was injected into the microcatheter through a microinjection pump (Cetoni GmbH). The microcatheter loaded with alginate solution was placed in a CaCl_2_ (10043‐52‐4, Sigma‐Aldrich) solution with a concentration of 200 mm for 30 s, and then taken out and placed in DI water for storage. The release of cargo from the Microcatheter robot was achieved by the degradation of alginate. The enzyme (Alginate Lyase, 9024‐15‐1, Sigma‐Aldrich) was prepared with 1X phosphate buffered saline (PBS) at a mass ratio of 1%. The microcatheter robot was placed in an environment of 37 °C and the enzymatic degradation solution was added to make the enzyme concentration (10^−8^ mg mL^−1^) stable in the overall environment. Immediately, the sample was placed under a fluorescence microscope and photographed, mainly using Fluorescein Isothiocyanate (FITC) and Bright filter, and photographed every 5 min for 24 h.

### Microcatheter Connects with Medical Tubing

One end of the microcatheter was coated with a biocompatible UV‐curable adhesive (Loctite AA 3942, Henkel), connected to one end of the medical tubing (*ID* = 0.38 mm, polyurethane medical tubing, Nordson Medical), and irradiated with a UV lamp for 1 min at a wavelength of 365 nm and a power of 100 mW cm^−2^.

### Magnetic Actuation and Demonstration

In the deflection demonstration experiment for the guiding microcatheter, a uniform magnetic field was applied using a pair of Helmholtz coils. The guiding microcatheter was positioned vertically at the center of the coils, where it initially drooped due to gravity. Upon applying the magnetic field, magnetic torque induced deflection in the microcatheter, and by modulating the local stiffness of the guiding microcatheter, different spatial deflection ranges were achieved.

(1) $$T_{\mathrm{mag}}=m\times B$$

Here *T*_mag_ is the magnetic torque, m is the magnetic dipole moment, B is the strength of the external magnetic field generated by the coils. Copper wires of various lengths were inserted into the guiding microcatheter. To ensure smooth insertion and reduce both friction and the risk of puncture, the wire tips were carefully polished, and plasma treated. Polishing was performed using a gradient of sandpaper (P1200–P7000), with the wires cleaned using isopropyl alcohol throughout the process. After drying, the wires underwent plasma treatment (99 watts, 1 min) to create a hydrophilic surface and were wetted prior to insertion. The position of the guiding microcatheter tip was recorded under different magnetic field strengths, and a sequence overlay was generated to visualize the resulting displacement (Figure 3D). For the navigation and crawling demonstrations proposed in this article, cylindrical NdFeB magnets (STM‐10x50‐N‐D, magnets4you GmbH) with a diameter of 10 mm and a length of 50 mm were used to apply the magnetic field required for long‐distance crawling. For magnetic steering and navigation, the magnets were manually manipulated to change their position and direction, and the magnets were kept at the proximal end of the guiding microcatheter when the guiding microcatheter was advanced, thereby changing the direction and strength of the applied magnetic field. For crawling, the position and direction of the magnet need to be manually manipulated, and the magnet speed can be changed by changing the parameters of the stepper motor driver controller (GY20512‐FBA, PEMENOL). When the microcatheter robot is connected to the medical tube for crawling, the magnet needs to be manually manipulated to keep it at the proximal end of the microcatheter robot, and the entire body is advanced by pushing the distal end combined with the crawling of the proximal end, and the steering and navigation tasks are controlled by visual feedback.

### Ex Vivo Experiments

The mouse uteri used in this article were obtained from mice sacrificed by the Transgenic Core Facility of the Max Planck Institute of Molecular Cell Biology and Genetics (Dresden) under license numbers TVV 5 712 021 (Etablierung einer neuartigen, nicht‐invasiven Methods für den Embryotransfer mit innovativen Mikrorobotik‐Tools). The mouse uteri used for ultrasound imaging (US imaging) were removed after mouse sacrifice, placed in PBS, stored at 5 °C, and used within 24 h. In the first ex vivo experiment, the mouse uterus was placed directly on the surface of acrylic support covered with ultrasound gel (Aquasonic) for signal transduction and directly subjected to ultrasound imaging. In the second protocol, the mouse uterus was placed on the surface of agar and covered with ultrasound gel on top. In the last protocol, the mouse uterus was placed inside agar, covered with ultrasound gel on the surface of agar, and subjected to ultrasound imaging. 100 mL of DI water was heated to 80 °C, and then 3 g of agarose was slowly added and gently mixed to form a homogenous solution (3% wt). A microtube with an outer diameter of about 400 µm was inserted into the mouse uterus for support. After it was placed in a container, an agarose solution at about 40 °C was poured in. After cooling to room temperature and becoming a gel, the microtube was taken out. The magnetic drive of the microcatheter robot was achieved using a magnet placed under the phantom, with a rotation frequency of 7 Hz and a magnetic field strength of about 100 mT. At the same time, to visualize such microcatheter robots within the tissue using existing imaging technology, a US imaging system (FUJIFILM VisualSonics) was used in the experiment. The experiment was conducted using a 256‐element transducer with a center frequency of 25–57 MHz, and the captured data was collected and reconstructed using onboard postprocessing.

### In Vivo Experiments

The mice used in this article were obtained from the Transgenic Core Facility of the Max Planck Institute of Molecular Cell Biology and Genetics (Dresden) under licenses TVV 5 712 021 (Etablierung einer neuartigen, nicht‐invasiven Methoden für den Embryotransfer mit innovativen Mikrorobotik‐Tools). They comply with the guidelines of the Federation of European Laboratory Animal Science Associations (FELASA) and the National Law on Laboratory Animal Experiments (Law No. 18.611). Twelve‐week‐old mice were used for in‐utero imaging. All mice were anesthetized with 1.5%–2% isoflurane. O_2_ flow (maintained at 1 − 2 mL min^−1^) was mixed with isoflurane. To optimize the coupling of ultrasound gel, the hair on the abdomen of the mice was removed using a commercially available depilatory cream. Ultrasound imaging was also performed using a US imaging system. The experiment was conducted using a 256‐element transducer with a center frequency of 25–57 MHz. After finding the mouse uterus in the ultrasound imaging system, the robot entered the mouse body through the vagina under the drive and navigation of the magnet. When the tip of the long microcatheter robot reached the bottom of the uterus, the crawling drive was stopped, and a microfluidic pump (Cetoni GmbH) was used to deliver blue dye (6104‐58‐1, Sigma‐Aldrich) to the inside of the uterus through the medical tubing to the long microcatheter robot. The magnet was reversely controlled to rotate and drive the long microcatheter robot to exit the uterus.

## Conflict of Interest

The authors declare no conflict of interest.

## Supporting information



Supporting Information

Supplemental Video 1

Supplemental Video 2

Supplemental Video 3

Supplemental Video 4

Supplemental Video 5

Supplemental Video 6

## Data Availability

The data that support the findings of this study are available from the corresponding author upon reasonable request.

## References

[1] K. Fuchs , Endoscopy 2002, 34, 154.11822011

[2] G. V. Rao , M. J. Mansard , P. K. Ravula , P. Rebala , R. R. Dama , D. N. Reddy , Asian J. Endosc. Surg. 2009, 2, 56.

[3] S. Atallah , B. Martin‐Perez , D. Keller , J. Burke , L. Hunter , Br. J. Surg. 2015, 102, 73. 10.1002/bjs.9710 25627137

[4] K. H. Dellimore , S. E. Franklin , A. R. Helyer , Cardiovasc. Eng. Tech. 2014, 5, 217.

[5] M. J. Grundeken , X. Li , C. E. Kurpershoek , M. C. Kramer , A. Vink , J. J. Piek , J. G. Tijssen , K. T. Koch , J. J. Wykrzykowska , R. J. de Winter , Circ. Cardiovasc. Interv. 2015, 8, 001816. 10.1161/CIRCINTERVENTIONS.114.001816 25582439

[6] M. E. Abdelaziz , L. Tian , M. Hamady , G.‐Z. Yang , B. Temelkuran , Prog. Biomed. Eng. 2021, 3, 032004.

[7] M. Keenan , T. H. Tate , K. Kieu , J. F. Black , U. Utzinger , J. K. Barton , Biomed. Opt. Express 2017, 8, 124. 28101406 10.1364/BOE.8.000124 PMC5231286

[8] R. Cordova , K. Kiekens , S. Burrell , W. Drake , Z. Kmeid , P. Rice , A. Rocha , S. Diaz , S. Yamada , M. Yozwiak , O. L. Nelson , G. C. Rodriguez , J. Heusinkveld , I. M. Shih , D. S. Alberts , J. K. Barton , J. Biomed. Opt. 2021, 26, 076001. 34216135 10.1117/1.JBO.26.7.076001 PMC8253554

[9] A. D. Rocha , W. K. Drake , P. F. Rice , D. J. Long , H. Shir , R. H. M. Walton , M. N. Reed , D. Galvez , T. Gorman , J. M. Heusinkveld , J. K. Barton , J. Biomed. Opt. 2023, 28, 121206. 37577082 10.1117/1.JBO.28.12.121206 PMC10423010

[10] T. H. Tate , M. Keenan , J. Black , U. Utzinger , J. K. Barton , J. Biomed. Opt. 2017, 22, 036013. 28334332 10.1117/1.JBO.22.3.036013 PMC5363790

[11] A. Chalazonitis , I. Tzovara , F. Laspas , P. Porfyridis , N. Ptohis , G. Tsimitselis , Curr. Probl. Diagn. Radiol. 2009, 38, 199. 19632497 10.1067/j.cpradiol.2008.02.003

[12] I. Roest , N. van Welie , V. Mijatovic , K. Dreyer , M. Bongers , C. Koks , B. W. Mol , Hum. Reprod. Open. 2020, 2020, hoz045. 31976383 10.1093/hropen/hoz045 PMC6964222

[13] M. Jain , M. Singh , Assisted Reproductive Technology (ART) Techniques, StatPearls Publishing, Treasure Island, FL 2025. 35015434

[14] B. M. Carlson , in Human Embryology and Developmental Biology, (5th ed.), (Eds: B. M. Carlson , W. B. Saunders ), Elsevier, Philadelphia 2014.

[15] T. Abyholm , T. Tanbo , Curr. Opin. Obstet. Gynecol. 1993, 5, 615. 8241437

[16] E. Mor , M. Vermesh , Fertil. Steril. 2005, 83, S27.

[17] Y. Chen , S. Waseem , L. Luo , Pathol. Res. Pract. 2025, 266, 155813. 39808858 10.1016/j.prp.2025.155813

[18] M. Al‐Jefout , Adv. Gynecol. Endosc. 2011, 23, 183.

[19] G. J. Criner , R. Eberhardt , S. Fernandez‐Bussy , D. Gompelmann , F. Maldonado , N. Patel , P. L. Shah , D.‐J. Slebos , A. Valipour , M. M. Wahidi , M. Weir , F. J. Herth , Am. J. Respir. Crit. Care. Med. 2020, 202, 29. 32023078 10.1164/rccm.201907-1292SO

[20] M. Russo , S. M. H. Sadati , X. Dong , A. Mohammad , I. D. Walker , C. Bergeles , K. Xu , D. A. Axinte , Adv. Intell. Syst. 2023, 5, 2200367.

[21] W. Yang , Y. Cao , S. Wang , Z. Liu , H. Cheng , L. Xie , Int. J. Comput. Assist. Radiol. Surg. 2024, 19, 1809. 38809318 10.1007/s11548-024-03194-z

[22] R. Zhang , D. Xie , C. Qian , X. Duan , C. Li , Robotica 2023, 41, 1055.

[23] M. Jolaei , A. Hooshiar , J. Dargahi , M. Packirisamy , Soft Robot 2021, 8, 340. 32678722 10.1089/soro.2020.0006

[24] M. Mattmann , C. De Marco , F. Briatico , S. Tagliabue , A. Colusso , X.‐Z. Chen , J. Lussi , C. Chautems , S. Pané , B. Nelson , Adv. Sci. 2022, 9, 2103277. 10.1002/advs.202103277 PMC8728812 34723442

[25] Y. Hu , W. Li , L. Zhang , G.‐Z. Yang , IEEE Robot. Autom. Lett. 2019, 4, 1669.

[26] H. Clogenson , J. Dankelman , J. Van Den Dobbelsteen , J. Med. Devices 2014, 8, 021002.

[27] T. Couture , J. Szewczyk , J. Med. Devices 2018, 12, 011003.

[28] T. da Veiga , J. H. Chandler , P. Lloyd , G. Pittiglio , N. J. Wilkinson , A. K. Hoshiar , R. A. Harris , P. Valdastri , Prog. Biomed. Eng. 2020, 2, 032003.

[29] T. Gopesh , J. H. Wen , D. Santiago‐Dieppa , B. Yan , J. S. Pannell , A. Khalessi , A. Norbash , J. Friend , Sci. Robot. 2021, 6, abf0601. 10.1126/scirobotics.abf0601 PMC9809155 34408094

[30] Z. Yang , H. Yang , Y. Cao , Y. Cui , L. Zhang , Adv. Intell. Syst. 2023, 5, 2200416.

[31] Y. Kim , G. A. Parada , S. Liu , X. Zhao , Sci. Robot. 2019, 4, aax7329. 10.1126/scirobotics.aax7329 33137788

[32] L. Pancaldi , L. Noseda , A. Dolev , A. Fanelli , D. Ghezzi , A. J. Petruska , M. S. Sakar , Adv. Intell. Syst. 2022, 4, 2100247.

[33] D. Chathuranga , P. Lloyd , J. H. Chandler , R. A. Harris , P. Valdastri , IEEE Robot. Autom. Lett. 2024, 9, 183.

[34] Y. Yan , T. Wang , R. Zhang , Y. Liu , W. Hu , M. Sitti , Sci. Adv. 2023, 9, adi3979. 10.1126/sciadv.adi3979 PMC10431716 37585531

[35] R. Dreyfus , Q. Boehler , S. Lyttle , P. Gruber , J. Lussi , C. Chautems , S. Gervasoni , J. Berberat , D. Seibold , N. Ochsenbein‐Kölble , M. Reinehr , M. Weisskopf , L. Remonda , B. J. Nelson , Sci. Robot. 2024, 9, adh0298. 10.1126/scirobotics.adh0298 38354258

[36] V. Iacovacci , E. Diller , D. Ahmed , A. Menciassi , Annu. Rev. Biomed. Eng. 2024, 26, 561. 38594937 10.1146/annurev-bioeng-081523-033131

[37] B. J. Nelson , I. K. Kaliakatsos , J. J. Abbott , Annu. Rev. Biomed. Eng. 2010, 12, 55. 20415589 10.1146/annurev-bioeng-010510-103409

[38] X. Arqué , M. D. T. Torres , T. Patiño , A. Boaro , S. Sánchez , C. de la Fuente‐Nunez , ACS Nano 2022, 16, 7547. 35486889 10.1021/acsnano.1c11013 PMC9134509

[39] V. Magdanz , S. Sanchez , O. G. Schmidt , Adv. Mater. 2013, 25, 6581. 23996782 10.1002/adma.201302544

[40] J. Kim , P. Mayorga‐Burrezo , S.‐J. Song , C. C. Mayorga‐Martinez , M. Medina‐Sánchez , S. Pané , M. Pumera , Chem. Soc. Rev. 2024, 53, 9190. 39139002 10.1039/d3cs00777d

[41] M. Medina‐Sánchez , L. Schwarz , A. K. Meyer , F. Hebenstreit , O. G. Schmidt , Nano Lett. 2016, 16, 555. 26699202 10.1021/acs.nanolett.5b04221

[42] C. Simó , M. Serra‐Casablancas , A. C. Hortelao , V. Di Carlo , S. Guallar‐Garrido , S. Plaza‐García , R. M. Rabanal , P. Ramos‐Cabrer , B. Yagüe , L. Aguado , L. Bardia , S. Tosi , V. Gómez‐Vallejo , A. Martín , T. Patiño , E. Julián , J. Colombelli , J. Llop , S. Sánchez , Nat. Nanotechnol. 2024, 19, 554. 38225356 10.1038/s41565-023-01577-y PMC11026160

[43] Z. Chen , M. M. Sánchez , Nat. Rev. Electr. Eng. 2024, 1, 759.

[44] M. Medina‐Sánchez , O. G. Schmidt , Nature 2017, 545, 406. 28541344 10.1038/545406a

[45] S. Jeon , S. Kim , S. Ha , S. Lee , E. Kim , S. Y. Kim , S. H. Park , J. H. Jeon , S. W. Kim , C. Moon , B. J. Nelson , J.‐y. Kim , S.‐W. Yu , H. Choi , Sci. Robot. 2019, 4, aav4317. 10.1126/scirobotics.aav4317 33137727

[46] F. Soto , E. Karshalev , F. Zhang , B. Esteban Fernandez de Avila , A. Nourhani , J. Wang , Chem. Rev. 2021, 122, 5365. 33522238 10.1021/acs.chemrev.0c00999

[47] R. Nauber , S. R. Goudu , M. Goeckenjan , M. Bornhäuser , C. Ribeiro , M. Medina‐Sánchez , Nat. Commun. 2023, 14, 728. 36759511 10.1038/s41467-023-36215-7 PMC9911761

[48] J. Sikorski , S. Mohanty , S. Misra , IEEE Robot. Autom. Lett. 2020, 5, 5260.

[49] B. Wang , K. F. Chan , K. Yuan , Q. Wang , X. Xia , L. Yang , H. Ko , Y.‐X. J. Wang , J. J. Y. Sung , P. W. Y. Chiu , L. Zhang , Sci. Robot. 2021, 6, abd2813. 10.1126/scirobotics.abd2813 34043547

[50] H. Torlakcik , S. Sevim , P. Alves , M. Mattmann , J. Llacer‐Wintle , M. Pinto , R. Moreira , A. D. Flouris , F. C. Landers , X.‐Z. Chen , J. Puigmartí‐Luis , Q. Boehler , T. S. Mayor , M. Kim , B. J. Nelson , S. Pané , Adv. Sci. 2024, 11, 2404061. 10.1002/advs.202404061 PMC11481240 39119930

[51] L. Hertle , S. Sevim , J. Zhu , V. Pustovalov , A. Veciana , J. Llacer‐Wintle , F. C. Landers , H. Ye , X.‐Z. Chen , H. Vogler , U. Grossniklaus , J. Puigmartí‐Luis , B. J. Nelson , S. Pané , Adv. Mater. 2024, 36, 2402309. 10.1002/adma.202402309 38780003

[52] V. Iacovacci , L. Ricotti , E. Sinibaldi , G. Signore , F. Vistoli , A. Menciassi , Adv. Sci. 2018, 5, 1800807. 10.1002/advs.201800807 PMC6145422 30250809

[53] M. L. Eisenberg , S. C. Esteves , D. J. Lamb , J. M. Hotaling , A. Giwercman , K. Hwang , Y.‐S. Cheng , Nat. Rev. Dis. Primers 2023, 9, 49. 37709866 10.1038/s41572-023-00459-w

[54] M. Punab , O. Poolamets , P. Paju , V. Vihljajev , K. Pomm , R. Ladva , P. Korrovits , M. Laan , Hum. Reprod. 2017, 32, 18. 27864361 10.1093/humrep/dew284 PMC5165077

[55] F. H. Biase , S. E. Moorey , J. G. Schnuelle , S. Rodning , M. S. Ortega , T. E. Spencer , PNAS Nexus 2023, 2, pgad284. 37711857 10.1093/pnasnexus/pgad284 PMC10498941

[56] M. Enciso , J. Aizpurua , B. Rodríguez‐Estrada , I. Jurado , M. Ferrández‐Rives , E. Rodríguez , E. Pérez‐Larrea , A. B. Climent , K. Marron , J. Sarasa , Sci. Rep. 2021, 11, 13420. 34183760 10.1038/s41598-021-92955-w PMC8238935

[57] R. Zhao , Y. Kim , S. A. Chester , P. Sharma , X. Zhao , J. Mech. Phys. Solids. 2019, 124, 244.

[58] R. Fujiwara , T. Devillers , D. Givord , N. M. Dempsey , AIP Adv. 2018, 8, 056225.

[59] M. Ghezelbash , S. M. R. Darbani , A. E. Majd , A. Ghasemi , J. Supercond. Nov. Magn. 2017, 30, 1893.

[60] C. Chautems , A. Tonazzini , Q. Boehler , S. H. Jeong , D. Floreano , B. J. Nelson , Adv. Intell. Syst. 2020, 2, 1900086.

[61] L. Schwarz , D. D. Karnaushenko , F. Hebenstreit , R. Naumann , O. G. Schmidt , M. Medina‐Sánchez , Adv. Sci. 2020, 7, 2000843. 10.1002/advs.202000843 PMC7509649 32999835

[62] X. Song , Z. Chen , X. Zhang , J. Xiong , T. Jiang , Z. Wang , X. Geng , U. K. Cheang , Sci. Rep. 2021, 11, 7907. 33846437 10.1038/s41598-021-87010-7 PMC8041914

[63] W. Gao , S. Sattayasamitsathit , A. Uygun , A. Pei , A. Ponedal , J. Wang , Nanoscale 2012, 4, 2447. 22374514 10.1039/c2nr30138e

[64] Z. Ren , R. Zhang , R. H. Soon , Z. Liu , W. Hu , P. R. Onck , M. Sitti , Sci. Adv. 2021, 7, abh2022. 10.1126/sciadv.abh2022 PMC8245043 34193416

[65] M. Thiriet , in PanVascular Medicine, (Ed: P. Lanzer ), Springer, Berlin 2015.

[66] S. Li , D. Liu , Y. Hu , Z. Su , X. Zhang , R. Guo , D. Li , Y. Lu , ACS Appl. Mater. Interfaces. 2022, 14, 10856. 35188736 10.1021/acsami.1c25102

[67] R. A. Lyons , E. Saridogan , O. Djahanbakhch , Hum. Reprod. Update. 2006, 12, 363. 16565155 10.1093/humupd/dml012

[68] E. C. W. Group , Hum. Reprod. Update. 2005, 11, 261. 15831503 10.1093/humupd/dmi006

[69] S. Texture , https://www.fab.com/listings/58e4d610‐454d‐450e‐9057‐b01a7415b635 (accessed: August 2024).

[70] B. Rivkin , C. Becker , B. Singh , A. Aziz , F. Akbar , A. Egunov , D. D. Karnaushenko , R. Naumann , R. Schäfer , M. Medina‐Sánchez , D. Karnaushenko , O. G. Schmidt , Sci. Adv. 2021, 7, abl5408. 10.1126/sciadv.abl5408 PMC8682992 34919439

[71] A. Ovassapian , Clin. Chest Med. 2001, 22, 281. 11444112 10.1016/s0272-5231(05)70043-5

[72] H. Hautmann , A. Schneider , T. Pinkau , F. Peltz , H. Feussner , Chest 2005, 128, 382. 16002960 10.1378/chest.128.1.382

[73] A. Bush , S. Cunningham , J. de Blic , A. Barbato , A. Clement , R. Epaud , M. Hengst , N. Kiper , A. G. Nicholson , M. Wetzke , D. Snijders , N. Schwerk , M. Griese , Thorax 2015, 70, 1078. 26135832 10.1136/thoraxjnl-2015-207349

[74] C. Hagberg , R. Georgi , C. Krier , Best Pract. Res. Clin. Anaesthesiol. 2005, 19, 641. 16408539 10.1016/j.bpa.2005.08.002

[75] M. Brewin , M. Birch , D. Mehta , J. Reeves , S. Shaw , C. Kruse , J. Whiteman , S. Hu , Z. Kenz , H. Banks , Ann. Biomed. Eng. 2015, 43, 2587. 25773982 10.1007/s10439-015-1294-7

[76] G. R. Cunha , A. Sinclair , W. A. Ricke , S. J. Robboy , M. Cao , L. S. Baskin , Differentiation 2019, 110, 49. 31622789 10.1016/j.diff.2019.07.004 PMC7339118

[77] C. M. Heunis , Y. P. Wotte , J. Sikorski , G. P. Furtado , S. Misra , IEEE Robot. Autom. Lett. 2020, 5, 705.

[78] J. W. Martin , B. Scaglioni , J. C. Norton , V. Subramanian , A. Arezzo , K. L. Obstein , P. Valdastri , Nat. Mach. Intell. 2020, 2, 595. 33089071 10.1038/s42256-020-00231-9 PMC7571595

[79] Y. Kim , E. Genevriere , P. Harker , J. Choe , M. Balicki , R. W. Regenhardt , J. E. Vranic , A. A. Dmytriw , A. B. Patel , X. Zhao , Sci. Robot. 2022, 7, abg9907. 10.1126/scirobotics.abg9907 PMC9254892 35417201

[80] R. Li , J. Wang , X. Zhao , Z. Liu , P. Jia , Y. Liu , G. Lin , H. Xu , J. Xiong , Biosens. Bioelectron. 2025, 270, 116977. 39586145 10.1016/j.bios.2024.116977

[81] X. Jin , S. Guo , J. Guo , P. Shi , T. Tamiya , H. Hirata , IEEE Sens. J. 2021, 21, 12284.

[82] S. Pane , M. Zhang , V. Iacovacci , L. Zhang , A. Menciassi , APL Bioeng. 2022, 6, 036102. 35935094 10.1063/5.0097145 PMC9348897

[83] C. Dillinger , C. Dillinger , A. Rasaiah , A. Vogel , C. Bahou , K. Monastyrskaya , A. H. Gheinani , D. Ahmed , Sci. Adv. 2025, 11, eadt8887. 40680140 10.1126/sciadv.adt8887 PMC12273784

[84] F. Fischer , M. Jeong , T. Qiu , Appl. Phys. Lett. 2024, 125, 074102.

[85] W. Xi , F. Kong , J. C. Yeo , L. Yu , S. Sonam , M. Dao , X. Gong , C. T. Lim , Proc. Natl. Acad. Sci. USA 2017, 114, 10590. 28923968 10.1073/pnas.1712195114 PMC5635922

[86] I. V. Soldatov , R. Schäfer , Rev. Sci. Instrum. 2017, 88, 073701. 28764508 10.1063/1.4991820

[87] D. Makarov , D. D. Sheka , Curvilinear Micromagnetism: From Fundamentals to Applications, Vol. 146, Springer Cham, Zurich 2022.

