# Supplementary material for: Rapid Fabrication of Self‐Propelled and Steerable Magnetic Microcatheters for Precision Medicine
Source: Adv Mater. 2025 Nov 27;38(51):e06591. doi: 10.1002/adma.202506591 (PMC13568938; doi:10.1002/adma.202506591)
Supplement: Supplementary file 1 [file ADMA-38-e06591-s001.docx]

Supporting Information

Rapid Fabrication of Self-Propelled and Steerable Magnetic Microcatheters for Precision Medicine

Zhi Chen, Boris Rivkin, David Castellanos-Robles, Ivan Soldatov, Lukas Beyer, Mariana Medina Sánchez*

Zhi Chen, David Castellanos-Robles, Mariana Medina Sánchez

NanoBiosystems Group, CIC nanoGUNE, San Sebastián, Spain

Boris Rivkin, Ivan Soldatov, Lukas Beyer

Institute for Solid State and Materials Research (IFW), Dresden, Germany

David Castellanos-Robles, Mariana Medina Sánchez

Center for Molecular Bioengineering (B CUBE), Dresden University of Technology, Dresden, Germany

Lukas Beyer

Institute of Materials Science, Technische Universität Bergakademie Freiberg, Freiberg, Germany

Mariana Medina Sánchez

IKERBASQUE, Basque Foundation for Science, Bilbao, Spain.

*Corresponding author:

Dr. Mariana Medina-Sánchez (m.medina@nanogune.eu)

**List of acronyms and abbreviations**

3D	Three-dimensional

MIS	Minimally invasive surgery

HSG	Hysterosalpingography

RMN	Remote magnetic navigation

mCR	Magnetically driven continuum robot

mSCR	Magnetic soft continuum robot

TubeBot	The tubular microrobot

PDMS	Polydimethylsiloxane

NdFeB	Neodymium-iron-boron

SEM	Scanning electron microscopy

VSM	Vibrating sample magnetometry

MOKE	Magneto-optical Kerr effect

PBS	Phosphate-buffered saline

BOECs	Bovine oviduct epithelial cells

PEEK	Polyetheretherketone

TPU	Thermoplastic polyurethane

US	Ultrasound

MicroCT	Micro Computed Tomography

**Supplementary Note:**

**Proof-of-concept Fabrication with Ecoflex**

To explore the versatility of our fabrication platform, we performed a proof-of-concept experiment using another highly flexible silicone, Ecoflex™ 00-30. Unlike the PDMS, Ecoflex is a room-temperature curing silicone. Use the same preparation process as the "Preparation of Magnetic Microcatheter" section. By carefully adjusting the process parameters of the Joule heating device, Ecoflex resin was able to be cured on a tungsten wire without causing significant thermal degradation. The applied voltage was 7 V, the current was 2.15 A, and the tungsten filament pulling speed was 2 mm/s. The process successfully generated hollow, flexible microtube structures. As shown in Figure S2. While further optimization is ongoing, these initial results effectively demonstrate the platform's versatility in processing materials such as Ecoflex. However, the system is primarily designed for processing thermocurable polymers.

**Landau-Levich-Derjaguin theory**

According to the Landau-Levich-Derjaguin theory,^[1]^

$$h=\frac{1.34r{Ca}^{\frac{2}{3}}}{1-1.34{Ca}^{\frac{2}{3}}}$$

where h is the thickness of the resin mixture coating, which corresponds to the wall thickness of the microcatheter after curing. r is the radius of the tungsten wire, Ca is the capillary number,

$$Ca=\frac{\mu V}{\sigma}$$

with $\mu$ as the dynamic viscosity of the resin mixture, V the pulling speed and $\sigma$ the surface tension of the resin. Due to the outer diameter of the microcatheter$(d=2(r+h))$, the outer diameter of the microcatheter is,

$$d=2r\left( 1+\frac{1.34\left( \frac{\mu V}{\sigma} \right)^{\frac{2}{3}}}{1-1.34\left( \frac{\mu V}{\sigma} \right)^{\frac{2}{3}}} \right)$$

Therefore, when the properties of the resin mixture remain unchanged, the outer diameter of the microcatheter is positively correlated with the pulling speed of the tungsten wire.

**TubeBot testing in BOECs model**

The bovine oviducts were obtained from a slaughterhouse (Vorwerk Podemus e.K., Dresden, Germany). Bovine oviduct epithelial cells (BOECs) were isolated and cultured according to the procedure described by Palma-Vera et al..^[2]^ Surrounding tissues and blood vessels were removed from the oviducts, and they were cleaned with Dulbecco PBS, followed by filling the lumen with collagenase 1A (1 mg/mL in Ham’s F12 medium) for 1 hr at 37°C. The mucosa was then extruded and passed through a 40 μm pore-size filter. The remaining cell clusters on the filter were washed with PBS and incubated with 0.5% trypsin/EDTA at 37°C for 10 minutes. The collected cells were either seeded on an insert or cryopreserved. A total of $2\times{10}^{5}$ cells were seeded onto a 0.4 μm pore-size insert (Sarstedt 24-well plate) pre-coated with rat-tail collagen I for cell culture, under conditions of 37°C, 5% CO₂, and 20% O₂. Within one week, the cells were maintained under liquid-liquid conditions using proliferation medium to promote cell division. Subsequently, the medium was removed from inside the insert to create an air-liquid interface to allow for cell differentiation. The cells were cultured for at least four weeks before experiments, with the medium being replaced twice a week.

Once a stable BOECs layer was established, it was immersed in medium and placed on the bottom of a culture dish. Cured PDMS (10:1 mass ratio) was cut into strips, placed on the BOECs layer, and covered with a coverslip, forming an in vitro model. The TubeBot was introduced into the channel of this in vitro model, and its magnetic actuation was achieved using magnets placed beneath the model, with a rotation frequency of 7 Hz and a magnetic field strength of approximately 100 mT. After several back-and-forth crawling movements within the model channel, the BOECs layer was retrieved for live/dead staining. Live cells were stained green with SYBR 14, while dead cells were stained red with Propidium Iodide (PI). The staining solution was prepared according to the kit instructions (L7011, Invitrogen). A total of 5 μL SYBR 14 was added to the channel and incubated for 10 minutes, followed by 5 μL PI for another 10 minutes. The in vitro model was then washed with pre-warmed fresh medium to remove the fluorescence staining. Images were taken using a fluorescence microscope (AxioObserver, Zeiss). SYBR 14 was visualized using a green filter (emission wavelength: 518 nm, excitation wavelength: 489 nm), while PI was visualized using a red filter (emission wavelength: 636 nm, excitation wavelength: 493 nm).

**Figure S1. A schematic illustration of the microcatheter robot platform fabrication setup.** The setup comprises key components including the resin container, preload device fixtures, leveling device, translation stage, and retainer fixtures.

**Figure S2. Proof-of-concept fabrication of micro-tubular structures using Ecoflex™ 00-30.** (A) Schematic of the modified fabrication process using Ecoflex™ 00-30 silicone. (B) Optical microscope images of the resulting Ecoflex tube on the tungsten wire, showing its cross-section and surface. Bubbles start to appear during the polymerization of Ecoflex if there is an increase in the curing temperature. (C) Photograph of the final, free-standing and flexible Ecoflex catheters after being removed from the tungsten wire. (D) Time-lapse images demonstrate the functionality of the hollow channel by successfully transporting a dye solution through the Ecoflex catheter without showing any fluid leakage.

**Figure S3. Cross-sectional scanning electron microscope (SEM) images of the magnetic microcatheter.** (A) Distribution of NdFeB particles embedded in PDMS. (B) Magnified view showing individual NdFeB particle morphology.

**Figure S4. Magnetic hysteresis loops of the fabricated microcatheter samples**. (A) Magnetic properties of the microcatheter cured at elevated temperatures using the fabrication setup. (B) Magnetic properties of the microcatheter cured at room temperature. The circled regions highlight differences in coercivity and remanence between the two curing methods.

**Figure S5. AFM force measurements of the PDMS-NdFeB composite.** (A) Young’s modulus as a function of curing time, measured at a constant temperature (80 °C) and a matrix-to-curing-agent ratio of 10:1. (B) Young’s modulus as a function of curing temperature, with curing time fixed at 4 h and a 10:1 matrix-to-curing-agent ratio, showing a slight decrease in stiffness above 120 °C. (C) Young’s modulus as a function of the matrix-to-curing-agent ratio, indicating that higher curing agent concentrations lead to increased stiffness. (D) AFM-based DMT modulus map of the composite surface, illustrating the nanoscale spatial distribution of Young’s modulus.

**Figure S6. Magnetic navigation of the guiding microcatheter in a 2D maze model**. (A) Guiding microcatheter consisting of a microcatheter and medical tubing steered under a magnetic field. (B) The path used for navigation within the maze. (C) Sequential images illustrate the microcatheter's navigation through the maze at different times, demonstrating precise steering under magnetic control.

**Figure S7. Deflection range of the guiding microcatheter as a function of stiffness**. The overall stiffness of the guiding microcatheter was modulated by varying the length of the copper wire inserted into its working channel. Using a combination of a Helmholtz coil and a permanent magnet (0–100 mT), the microcatheter tip was steered along different trajectories to reach distinct target points in 3D space.

**Figure S8.** **Verification of microcatheter structural integrity after 20 wire insertion–retraction cycles**. (A) (i) Components used for the test: a microcatheter and copper wires (50 µm and 100 µm in diameter). (ii) Close-up image of the polished, rounded copper wire tip, designed to minimize the risk of internal damage during operation. (B) (i) Schematic and (ii) corresponding photograph illustrating the wire insertion and retraction process. (C) Microscopic images of the microcatheter body after 20 insertion/retraction cycles, showing no visible signs of wear or damage. (i) Image of one end of the microcatheter; (ii) image of the central section. (D) Fluid leakage test performed following cyclic loading. The images confirm the preservation of lumen integrity, with no fluid leakage observed during injection, thereby validating the microcatheter’s structural robustness.

**Figure S9. TubeBot magnetization analysis using magneto-optic Kerr effect (MOKE) microscopy.** (A) A schematic experiment, utilizing magneto-optical indicator film with perpendicular anisotropy magneto-optical indicator film with perpendicular anisotropy (PMOIF) positioned on top of a cylindrical microcatheter with altering magnetization vector orientation. (B) By using a magneto-optical wide-field Kerr microscope configured for polar sensitivity under low-resolution settings—where the individual garnet domains are not visible— a pole distribution within the microcatheter can be detected. (C) PMOIF images of the magnetization distribution within the microcatheter demonstrating the presence of nodes, indicating the altering magnetization along the TubeBot.

**Figure S10. TubeBot motion test.** The motion direction of TubeBot is the same as the rotation direction of the permanent magnet at different rotation frequencies.

**Figure S11. Velocity performance of the microcatheter robot.** (A) The crawling speed increases with increasing magnetic field rotation frequency, with a nearly linear relationship. (B) The crawling speed varies with the magnetization angle, reaching a peak at 45^o^. (C) Impact of different magnetization diameters, where asymmetrical magnetization results in varying speeds in different directions. (D) decreased crawling speed as microrobot length increases.

**Figure S12.** Navigation performance of TubeBots of varying lengths in a frequently angled channel. The plot shows the time required for TubeBots of different lengths to traverse the full channel along the same path. Markers indicate the positions of channel bends, and the corresponding time represents the duration from the start (0 s) to the moment each TubeBot passes that bend. All tests were conducted under a rotating magnetic field of 100 mT at 5 Hz. The results demonstrate that in a tortuous environment with continuously changing angles, shorter TubeBots achieve greater navigation efficiency.

**Figure S13.** Time-lapse images of TubeBots of varying lengths (L = 2, 4, 6, and 8 mm) navigating a channel with frequent directional changes. Each sequence captures the position and orientation of an individual TubeBot as it passes through a bend in the channel, along with the corresponding time.

**Figure S14. Different schemes TubeBot is magnetized.** (A) Maintaining the initial magnetization angle at 45°, microcatheters with different NdFeB particle contents were magnetized. The lengths were all 4 mm. (B) Microcatheters with NdFeB particle mass fraction of 50% were magnetized at different initial magnetization angles. The lengths were all 4 mm. (C) Maintaining the initial magnetization angle at 45°, microcatheters with different magnetization diameters were magnetized with a length of 4 mm. (D) Maintaining the same magnetization diameter and initial magnetization angle, microcatheters of different lengths were magnetized. (E) The mold for magnetization.

**Figure S15.** TubeBot navigating and crawling in a channel with variable diameter, with an image overlay showing the microrobot at different positions while moving along the channels. TubeBot navigating in path 2, with one end tracked to record movement over time.

**Figure S16.** TubeBot navigating and crawling in a channel with variable diameter. Image overlays show the microrobot at different positions as it moves along the channel, with velocity tracked. (A) TubeBot navigating in path 1. (B) TubeBot navigating in path 2.

**Figure S17. TubeBot climbs over steps in a variable diameter channel model.** It transitions from a smaller diameter to a larger diameter, and then from a larger diameter to a smaller diameter. (A) Top view. (B) Side view.

**Figure S18. The navigation crawling of the alginate-filled TubeBot in a complex channel model.** (A) The channel angle changes in sequence: 45°, 135°, 120°, 60°. (B) The channel is a straight line. (C) The channel angle changes in sequence: 40°, 140°, 120°, 60°. (D) The channel angle changes in sequence: 45°, 135°, 115°, 65°. (E) The channel angle changes in sequence: 55°, 125°, 115°, 65°. (F) The size of the complex channel model.

**Figure S19. TubeBot movement test after loading cargo.** (A) Deformation of alginate-filled TubeBot in a magnetic field (100mT). (B) Alginate-filled TubeBot crawling up and down in a sloping tube (8hz 100mt ID=1mm).

**Figure S20. TubeBot crawling on a bovine oviduct epithelial cell layer to assess the impact of movement on cell viability.** The driving magnetic field was set at 5 Hz and 100 mT. (A) Optical microscope image showing the TubeBot crawling forward on the cell layer. (B) Schematic illustration of the in vitro cell layer model. (C) Fluorescent live/dead staining images of the cell layer after the TubeBot crawled back and forth 10 times: (i) experimental sample, (ii) control sample. Live cells are stained green with SYBR 14 (excitation: 489 nm, emission: 518 nm), while dead cells are stained red with Propidium Iodide (PI) (excitation: 493 nm, emission: 636 nm).

**Figure S21. Stepped channel model.** (A) Image of the channel. (B) Dimensions of the channel.

**Figure S22. Demonstration of the microcatheter robot platform navigation through the 3D phantoms.** (A) Navigation and crawling of microcatheter robot in a 3D channel, shown in top view. The microcatheter robot enters the main channel (d = 3 mm) at t = 0 s, driven by a rotating magnetic field. At t = 20 s, the field is adjusted to guide it into side channel 1 (d = 1.5 mm) towards target point a. By t = 40 s, the microcatheter robot exits side channel 1, and at t = 50 s, it is guided to target point b in the main channel. At t = 100 s, the microcatheter robot enters side channel 2 (d = 1.5 mm) and reaches target point c. Finally, at t = 130 s, the microcatheter robot exits the main channel through a combination of the rotating magnetic field and manual pulling. (B) Navigation and crawling of the microcatheter robot in a full-scale 3D bronchial tree phantom, shown in top view. The microcatheter robot reaches the bronchial site at t = 25 s, enters the left bronchial tree at t = 49 s, and reaches target point a. By t = 102 s, it advances to target point b, and by t = 121 s, it reaches target point c. At t = 160 s, the microcatheter robot exits the left bronchial tree, enters the right bronchial tree, and reaches target point d. Finally, the microcatheter robot exits the bronchial tree at t = 184 s, driven by the rotating magnetic field and manual pulling.

**Figure S23. Two navigation methods to drive the microcatheter robot in a 3D tubing phantom:** A combined manual push and magnetic field guidance, as well as a combined manual push and magnetic field guidance and propulsion in (A) Narrow and sharp channels (2D), and (B) Upward and sharp channel (3D).

**Figure S24. *Ex vivo* navigation of the microcatheter robot in tissues under ultrasound guidance.** (A) US imaging of the microcatheter robot in uterine tissue ex vivo. (i) Schematic showing uterine tissue positioned on the surface (reproductive tract tissue) of an agar phantom. (ii) US imaging of the microcatheter robot crawling through the uterus on the agar phantom, maintaining its 3D structure. (B) (i) Schematic and (ii) time-lapse images demonstrating targeted deflection and positioning of the microcatheter robot within the rat lung tissue. (C) (i) Schematic and (ii) time-lapse ultrasound images showing the microcatheter navigating through an explanted pig trachea.

**Figure S25. Practical setups of *ex vivo* and *in vivo* experiments with integrated ultrasound imaging.** (A) *Ex vivo* tubing model. (B) *Ex vivo* reproductive tract tissue model with tissue immersed in PBS. (C) *Ex vivo* uterine horn tissue model embedded in agar. (D) Ex vivo pig trachea model. (E) *Ex vivo* rat lung model. (F) *In vivo* mouse model: mice anesthetized under continuous isoflurane, with physiological monitoring and heating to maintain body temperature. Ultrasound probe positioned above the reproductive system for imaging.

**Figure S26. The microcatheter robot for *in vivo* experiments.** The microcatheter robot is connected to medical tubing. One end of the medical tubing is connected to a syringe with blue dye inside.

**Figure S27. Trajectory time using different microcatheter advancement methods.** The hybrid method, which combines magnetic pushing, magnetic guidance, and wave-crawling propulsion, is faster at all angled sections. It cuts the total navigation time from 60 seconds down to 36 seconds, compared to using manual pushing and magnetic guidance (without wave-crawling propulsion).

**Figure S28.** **Magnetic navigation of the microcatheter robot in a 2D maze model.** (A) The microcatheter robot crawls under the action of the rotating magnetic field. (B) The path used for navigation within the maze. (C) Sequential images illustrate the microcatheter's navigation through the maze at different times, demonstrating precise navigation under magnetic control.

**Figure S29. Effect of wave-crawling microcatheter versus conventional push catheter on cell layer integrity in a cell-lined phantom channel.** (A) Image of the agar-based in vitro model and schematic diagram, showing a HeLa cell monolayer grown within an agar channel lined with a 0.4 µm pore size Polycarbonate Track-Etched (PCTE) membrane (Isopore™, Whatman, 10 mm diameter). (B) Time-lapse image of the microcatheter robot crawling through the cell monolayer channel, with 𝛼 and 𝛽 indicating subsequent fluorescently stained regions. (C) Time-lapse image of a conventional TPU catheter being pushed into the channel through the cell-coated area. (D) Quantitative XTT assay results comparing cell viability among control, crawling, and pushing groups. (E) Fluorescence images of HeLa monolayers stained with SYBR 14 (green, live cells) and propidium iodide (red, dead cells). The blue dashed line indicates the navigation path. (i) In the crawling group, the track (blue outline) is clearly visible, with minimal cell death. (ii) In the pushing group, movement causes a physical scratch, creating a cell-free void and edge-associated cell death. *(iii)* The control shows a confluent monolayer of mostly live cells with no disturbance.

**Table S1.** Comparison of submillimeter endoscopes and catheters for fallopian tube use.

| **Technology / System** | **Outer Diameter** | **Core Function** | **Propulsion Method** | **Advantages** | **Limitations / Risks** |
| --- | --- | --- | --- | --- | --- |
| Multispectral Imaging Endoscope^[3]^ | 0.8 mm | Multispectral fluorescence & reflectance imaging for early cancer detection. | External Push + Passive Tip Steering | Sub-millimeter size for natural orifice access; wide spectral range (250-642 nm) for detecting endogenous fluorescence. | Potential for UV light-induced tissue damage; reliance on passive insertion risks buckling or perforation; no therapeutic channel. |
| Combined OCT & Wide-Field Falloposcope^[4]^ | < 1.0 mm | Combined structural (OCT) and functional (wide-field) imaging for enhanced cancer detection. | External Push + Passive Tip Steering | Multimodal imaging improves diagnostic accuracy; sub-millimeter access to fallopian tube. | Fiber fragility: reliance on passive push forces for insertion carries risk of tissue damage; no therapeutic capability. |
| CAFE (Cell-Acquiring Fallopian Endoscope) System^[5]^ | ≤ 0.9 mm | Imaging (MFI/Reflectance) + Guided Cell Collection (scrape biopsy). | External Push + Passive Tip Steering | Combines diagnosis with sample collection; enables downstream cytological and -omics analyses. | Biopsy by scraping carries inherent risk of tissue damage; no active propulsion; working channel dedicated to custom wire, not general delivery. |
| Clinical MFI & OCT Falloposcope^[6]^ | ~0.8 mm | Multimodal diagnostic imaging (MFI & OCT) for clinical use. | External Push + Tip Steering | Clinically-validated, single-use, low-cost design (~$750/probe) with a portable system. | No therapeutic or biopsy channel; relies on passive insertion; failed to meet desired Field of View (FOV). |
| Self-Propelling Microcatheter Robot (This Work) | 350 - 400 µm | Active Navigation & Therapeutic Delivery (e.g., drugs, cells, embryos). | Magnetically-Actuated Wave-Crawling | Active self-propulsion reduces reliance on push forces, minimizing risk of buckling and perforation; significantly smaller diameter for less invasive access; designed as a delivery platform with a functional working channel. | Lack of haptic feedback; requires an external magnetic actuation system |

**Table S2.** Comparison of catheter designs: Fabrication, size, navigation, and applications.

| **Catheters** | **Fabrication** | **Size**  **[mm]** | **Navigation** | **Advancement** | **Adaptability** | ***In vitro/In vivo*** |
| --- | --- | --- | --- | --- | --- | --- |
| Ref. ^[7]^ | Mechanical assembly | 2 | Tendon | Mechanical push | No | *In vitro* |
| Ref. ^[8]^ | Mold casting | 0.9 | Hydraulic | Manual push | No | *In vitro* and *in vivo* |
| Ref. ^[9]^ | Mold casting | 1.3 | Shape memory alloy | Manual push | yes | *In vitro* |
| Ref. ^[10]^ | Mold casting | 1.67 | Magnet | Mechanical push | No | *In vitro* |
| Ref. ^[11]^ | Injection molding | 4–7 | Magnet | Mechanical push | No | *Ex vivo* and *in vivo* |
| Ref. ^[12]^ | Printing/injection molding | 0.5-0.8 | Magnet | Manual push | No | *In vitro* |
| Ref. ^[13]^ | Continuous curing | 0.35 | Magnet and flow | Flow and manual push | yes | *In vitro* and *ex vivo* |
| Ref. ^[14]^ | Injection molding | 2 | Magnet | Manual push | yes | *Ex vivo* and *in vivo* |
| Ref. ^[15]^ | Mechanical assembly | 0.7-0.8 | Magnet | Mechanical push and tip rotation | yes | *Ex vivo* and *in vivo* |
| This work | Continuous curing | 0.3-1 | Magnet | Manual push and end propulsion | yes | *Ex vivo* and *in vivo* |

**Table S3.** Comparison of representative tethered and untethered magnetic microrobots.

| **Feature** | **This Work's Microcatheter Robot** | **Representative untethered undulation/crawling soft robots ^[16]^** | **Tethered microrobot ^[17]^** | **Conventional Magnetic Catheters/Guidewire ^[18]^** |
| --- | --- | --- | --- | --- |
| Drive Principle | External rotating magnetic field for terminal wave-crawling | External magnetic field for overall body undulation/crawling | A rotating magnetic field generates undulations of the magnetic head, which pulls the tether | External magnetic field for tip steering |
| Tethered/  Untethered | Tethered, connected to medical tubing | Typically untethered | Tethered | Tethered |
| Size | Micron-scale, OD 0.3-1 mm | Millimeter to centimeter scale, or micron-scale with simpler structure | 0.4 mm x 0.2 mm | Millimeter scale/ Micron-scale |
| Working Channel | Yes, ID ~0.15 mm | No | No | Some may have |
| Primary Propulsion | Terminal active propulsion + manual push | Whole-body active locomotion | Active undulatory propulsion at the tip | Manual push/ Motor drive, magnetic steering assistance |
| Key Innovation/  Application Focus | “Moving without pushing”, terminal active propulsion for micron-scale tethered catheter, enhancing navigation in confined/complex paths, reducing tissue trauma, aiding in overcoming entrapment | Targeted delivery, micro-manipulation for untethered robots | Sensor-integrated device for autonomous navigation in sub-millimeter arteries using magnetic actuation | Improved tip maneuverability for conventional catheters |
| Specific Challenges Addressed | Force transmission loss, tip perforation risk, getting stuck in sharp turns for traditional microcatheters | Long-range precise control, in-body power supply, complex environment adaptation | Failure of flow-driven microcatheters to navigate in obstructed arteries with impaired blood flow | Balancing size, flexibility, and magnetic responsiveness |

**Table S4.** Comparison of the Joule heating with other representative microtube fabrication techniques

| **Feature** | **This Work** | **Molding ^[4]^** | **Dip-Coating ^[15]^** | **Coaxial Extrusion ^[16]^** | **Representative 3D Printing ^[17]^** |
| --- | --- | --- | --- | --- | --- |
| Principle | Joule heat from tungsten wire initiates continuous curing and pulling of resin | Material injected into a mold and cured | Mandrel dipped into material solution, then dried/cured | Core and shell materials extruded through concentric nozzles | Layer-by-layer material addition |
| Min. ID/Feature Size | Determined by tungsten wire diameter | Micron-scale possible, complex demolding | It depends on mandrel size and coating thickness control | Micron-scale possible, complex control | Micron-scale possible, speed/material limitations |
| Hollow Structure Capability | Yes, hollow channel formed after wire removal | Yes | Yes, after mandrel removal | Yes | Yes, strong capability for complex hollow designs |
| Material Compatibility (PDMS+Magnetic Particles) | Demonstrated good compatibility | Good, but need to concerned sedimentation problem | Good, but the solution viscosity needs control | Good, but material rheology matching needed | Feasible for some methods, but can be limited by viscosity/curing properties |
| Manufacturing Efficiency/Speed | Continuous process, potential for high efficiency/speed | Single piece or small batch, relatively slow | Multi-step process, moderate speed | Can be continuous, moderate to fast speed | Layer-by-layer, time-consuming for complex parts |
| Equipment Cost/Complexity | Relatively simple, lower cost | Mold design and fabrication have costs | Relatively simple equipment | More complex equipment, high control precision required | Higher equipment cost, complex maintenance |
| Scalability/Yield | Potentially high | Moderate, limited by mold number and cycle time | Moderate | Potentially high | Depends on printer size/number, low yield for single complex parts |
| Outer Diameter Control | Adjusting tungsten wire pulling speed, solution concentration, input Power | Fixed by mold dimensions | Number of dips/solution concentration | Nozzle dimensions/extrusion rates | CAD model design, but limited by printing range |
| Post-fabrication Magnetization | Yes | Yes | Yes | Yes | Yes, or some techniques support in-situ magnetization |

**Table S5.** Comparative analysis of catheter fabrication, cost, and end propulsion.

| **Catheters** | **Size**  **[mm]** | **Fabrication** | **Preparation Cost** | **End Propulsion** |
| --- | --- | --- | --- | --- |
| Ref. ^[19]^ | 2.6-6 | Mechanical assembly | High | Pneumatic Peristaltic Wave Propulsion |
| Ref. ^[20]^ | 108 | Mechanical assembly | High | Pneumatic Peristaltic Wave Propulsion |
| Ref. ^[21]^ | 25 | Mold casting and Mechanical assembly | High | Pneumatic Peristaltic Wave Propulsion |
| This work | 0.3-1 | Continuous curing | Low | Terminal undulatory crawling propulsion |

**Table S6.** Benchmark comparison between our microcatheter robot and representative research and commercial microcatheters.

| **Feature** | **This Work's microcatheter Robot** | **Soft robotic microcatheter**  **^[4]^** | **Helical microcatheter**  **^[11]^** | **Magnetically-guided microcatheter^[21]^** | **Rebar™ reinforced microcatheter (Medtronic)** | **Progreat™ Micro Catheter (Terumo)** |
| --- | --- | --- | --- | --- | --- | --- |
| Size OD/ID | 0.3-1 mm OD / 0.15 mm ID | 0.9 mm OD / 0.4 mm ID | 0.8mm OD / 0.17mm ID | 1.0 mm OD / 0.5 mm ID | ~2.7-2.8Fr OD (0.9-0.93mm) / 0.021"-0.027" ID (0.53-0.69mm) | ~1.7-2.8Fr OD (0.57-0.93mm) / 0.017"-0.027" ID (0.43-0.70mm) |
| Material | PDMS + NdFeB | Hyperelastic polymer | Stainless steel wire, nylon elastomer, NdFeB magnet | Tip: Silicone, Neodymium magnets. Body: Pebax, Stainless-steel spring, PTFE | Stainless-steel construction, PTFE inner liner, hydrophilic coating | Polyester elastomer, PTFE inner layer, hydrophilic coating M Coat |
| Manufacturing | Scalable Joule heating-based continuous curing | Mold casting | Laser welding, fusion, mechanical joining | Layer-by-layer assembly, thermal fusion, mechanical joining | Metal drawing process | Thermoplastic extrusion |
| Drive/Advancement | End-propelled wave-crawling assisted by manual push | Hydraulic actuation, manual push | Terminal screw propulsion, electric propulsion unit | Manual push with magnetic steering guidance | Manual push over guidewire | Manual push over guidewire |
| Flexibility/Compliance | Highly flexible PDMS matrix, stiffness tunable with Cu wire insertion | Flexible materials | Soft tip with gradient stiffness for balance of pushability and softness | Soft tip with graded stiffness for balance of flexibility and pushability | Semi-rigid proximal shaft, flexible distal shaft | Tip design for flexibility, braid for lumen integrity |
| Responsiveness | Responds to external rotating magnetic field for wave crawling propulsion；Bends in uniform field for steering | Responds to hydraulic input for actuation | Responds to external magnetic field for steering | Responds to external magnetic field for steering; Responds to syringe pressure for injection | Good response to guidewire manipulation and push force, steam-shapeable tip | Good response to guidewire manipulation and push force, various pre-shaped tips |
| Navigability | Demonstrated in tortuous channels, bronchial tree, *ex vivo* and *in vivo* mouse reproductive tract | *In vitro* and *in vivo* demonstrated | *In vitro* and *in vivo* demonstrated | In vitro demonstrated | Hydrophilic coating and kink resistance aid navigation in tortuous vessels | Hydrophilic coating and tip designs for branch selectivity |
| Working Channel | Yes, ~0.15 mm ID for fluid/drug delivery | Yes, ~0.4 mm ID for fluid delivery | Yes, 0.17 mm | Yes, ~0.5 mm ID | Yes, e.g., 0.021" or 0.027" | Yes, e.g., 0.017" to 0.027" |

**References**

[1]	D. Quéré, *Annu. Rev. Fluid Mech.* **1999**, 31, 347.

[2]	S. Palma-Vera, R. Einspanier, J. Schoen, *Reprod. Biol.* **2014**, 14, 206.

[3]	T. H. Tate, M. Keenan, J. Black, U. Utzinger, J. K. J. J. o. B. O. Barton, *J. Biomed. Opt.* **2017**, 22, 036013.

[4]	M. Keenan, T. H. Tate, K. Kieu, J. F. Black, U. Utzinger, J. K. Barton, *Biomed. Opt. Express* **2017**, 8, 124.

[5]	R. Cordova, K. Kiekens, S. Burrell, W. Drake, Z. Kmeid, P. Rice, A. Rocha, S. Diaz, S. Yamada, M. Yozwiak, O. L. Nelson, G. C. Rodriguez, J. Heusinkveld, I. M. Shih, D. S. Alberts, J. K. Barton, *J. Biomed. Opt.* **2021**, 26.

[6]	A. D. Rocha, W. K. Drake, P. F. Rice, D. J. Long, H. Shir, R. H. M. Walton, M. N. Reed, D. Galvez, T. Gorman, J. M. Heusinkveld, J. K. Barton, *J. Biomed. Opt.* **2023**, 28, 121206.

[7]	W. Yang, Y. Cao, S. Wang, Z. Liu, H. Cheng, L. Xie, *Int. J. Comput. Assist. Radiol. Surg.* **2024**, 19, 1809.

[8]	T. Gopesh, J. H. Wen, D. Santiago-Dieppa, B. Yan, J. S. Pannell, A. Khalessi, A. Norbash, J. Friend, *Sci. Robot.* **2021**, 6, eabf0601.

[9]	M. Mattmann, C. De Marco, F. Briatico, S. Tagliabue, A. Colusso, X. Z. Chen, J. Lussi, C. Chautems, S. Pané, B. Nelson, *Adv. Sci.* **2022**, 9, 2103277.

[10]	S. Jeon, A. K. Hoshiar, K. Kim, S. Lee, E. Kim, S. Lee, J.-y. Kim, B. J. Nelson, H.-J. Cha, B.-J. Yi, *Soft Robot.* **2019**, 6, 54.

[11]	C. Zhou, Y. Yang, J. Wang, Q. Wu, Z. Gu, Y. Zhou, X. Liu, Y. Yang, H. Tang, Q. Ling, L. Wang, J. Zang, *Nat. Commun.* **2021**, 12, 5072.

[12]	Y. Kim, G. A. Parada, S. Liu, X. Zhao, *Sci. Robot.* **2019**, 4, eaax7329.

[13]	L. Pancaldi, P. Dirix, A. Fanelli, A. M. Lima, N. Stergiopulos, P. J. Mosimann, D. Ghezzi, M. S. Sakar, *Nat. Commun.* **2020**, 11, 6356.

[14]	L. Mao, P. Yang, C. Tian, X. Shen, F. Wang, H. Zhang, X. Meng, H. Xie, *Nat. Commun.* **2024**, 15, 3759.

[15]	R. Dreyfus, Q. Boehler, S. Lyttle, P. Gruber, J. Lussi, C. Chautems, S. Gervasoni, J. Berberat, D. Seibold, N. Ochsenbein-Kölble, M. Reinehr, M. Weisskopf, L. Remonda, B. J. Nelson, *Sci. Robot.* **2024**, 9, eadh0298.

[16]	a)W. Hu, G. Z. Lum, M. Mastrangeli, M. Sitti, *Nature* **2018**, 554, 81; b)Z. Ren, R. Zhang, R. H. Soon, Z. Liu, W. Hu, P. R. Onck, M. Sitti, *Sci. Adv.* **2021**, 7, eabh2022.

[17]	L. Pancaldi, L. Noseda, A. Dolev, A. Fanelli, D. Ghezzi, A. J. Petruska, M. S. Sakar, *Adv. Intell. Syst.* **2022**, 4, 2100247.

[18]	a)C. Chautems, A. Tonazzini, Q. Boehler, S. H. Jeong, D. Floreano, B. J. Nelson, *Adv. Intell. Syst.* **2020**, 2, 1900086; b)S. Fu, B. Chen, D. Li, J. Han, S. Xu, S. Wang, C. Huang, M. Qiu, S. Cheng, X. Wu, L. Zhang, S. Du, T. Xu, *Adv. Intell. Syst.* **2023**, 5, 2300267; c)M. E. Tiryaki, Y. G. Elmacıoğlu, M. Sitti, *Sci. Adv.* **2023**, 9, eadg6438.

[19]	H. Tsukagoshi, K. Terashima, Y. Takai, presented at *2018 IEEE International Conference on Robotics and Automation (ICRA)*, 21-25 May 2018, **2018**.

[20]	H. Sato, K. Uchiyama, Y. Mano, F. Ito, S. Kurumaya, M. Okui, Y. Yamada, T. Nakamura, *IEEE Access* **2021**, 9, 165271.

[21]	T. Yamamoto, S. Sakama, A. Kamimura, *IEEE Robot. Autom. Lett.* **2020**, 5, 5034.
